# Pricing, Carbon Emission Reduction, Low-Carbon Promotion and Returning Decision in a Closed-Loop Supply Chain under Vertical and Horizontal Cooperation

**DOI:** 10.3390/ijerph14111332

**Published:** 2017-11-01

**Authors:** Hui Li, Chuanxu Wang, Meng Shang, Wei Ou

**Affiliations:** 1 School of Economics and Management, Shanghai Maritime University, No. 1550, Haigang Avenue, Pudong New Area, Shanghai 201306, China; 2 School of Flight, Anyang Institute of Technology, No. 1 Huanghe Avenue W., Anyang City 455000, China; shangmengkorea@gmail.com; 3 School of Knowledge Science, Japan Advanced Institute of Science and Technology, Asahidai 1-1, Nomi City, Ishikawa 923-1292, Japan; studyouwei@126.com

**Keywords:** carbon emissions reduction, low-carbon promotion, cooperation, closed-loop supply chain

## Abstract

In this paper, we examine the influences of vertical and horizontal cooperation models on the optimal decisions and performance of a low-carbon closed-loop supply chain (CLSC) with a manufacturer and two retailers, and study optimal operation in the competitive pricing, competitive the low-carbon promotion, the carbon emission reduction, the used-products collection and the profits. We consider the completely decentralized model, M-R vertical cooperation model, R-R horizontal cooperation model, M-R-R vertical and horizontal cooperation model and completely centralized model, and also identify the optimal decision results and profits. It can be observed from a systematic comparison and numerical analysis that the completely centralized model is best in all optimal decision results among all models. In semi-cooperation, the M-R vertical cooperation model is positive, the R-R horizontal cooperation model is passive, and the positivity of the M-R-R vertical and horizontal cooperation model decreases with competitive intensity increasing in the used-products returning, carbon emissions reduction level, low-carbon promotion effort and the profits of the manufacturer and the entire supply chain.

## 1. Introduction

In recent years, energy saving and environmental protection problems have been drawing the attention of global and national organizations. It is the most direct way to reduce the level of carbon emissions by solving the current social problems of saving energy and environmental protection. The collection and remanufacturing of used products (closed-loop supply chain—CLSC) can not only reduce the manufacturing cost of products and increase the profit of enterprises, but also reduce carbon emissions and environmental pollution. Motivated by the significance of CLSC management in practice, CLSC has also been a critical research topic in the academic area (Savaskan et al. [1]; Savaskan and Wassenhove [2]; Abbey et al. [3]). Manufacturers have realized that CLSC management could be used to gain competitive advantages and achieve sustainable development. In practice, some industries have implemented a reverse supply chain model with the collection channel of manufacturers. For example, in the copier manufacture and automobile industries, manufacturers have been leaders in reverse logistics and directly collect used products from customers. In recycling activities, some manufacturers also exert collection efforts such as product design, process modification to facilitate recycling, reverse logistics services, employee-training programs, etc. These activities reflect the environmentally responsible features of the firms and enhance their reputation, satisfying the environmental concerns of consumers and simplify their disposal process [4]. Besides economic and environmental benefits, manufacturers consider carbon emission reductions in the CLSC design.

Controlling the emissions of greenhouse gases has become a popular issue since the 1990s. The government, who is responsible for implementing carbon emission reduction policies, bears undeniable responsibilities for supervising the activities in this regard of manufacturing enterprises. Lots of countries have issued relevant policies about carbon emission reduction. No doubt this will bring about new standards and requirements for manufacturing enterprises. The American company Apple Inc. has employed the product life cycle analysis method to assess the carbon emission impact of its products. According to this study in 2011, Apple Inc. released 2310 tons of greenhouse gases, 61% of which were from the production processes of its products. Through increasing the recyclability and recycling efficiency of products, the Apple’s revenue has risen steadily during the past several years, while the greenhouse gas emissions per dollar revenue decreased by 15.4%. The Chinese government has issued its 13th Five-Year Plan for Conserving Energy and Reducing Emissions, which put forward new requirements for China’s pollution reduction activities, and energy conservation and emission reduction tasks of enterprises will be a more important part of the plans. Advertisements, as a marketing method, which can be used to establish and maintain a brand image, can increase its popularity as well as the reputation. They could also be used to increase the awareness of customers to the products that would achieve the brand transition among them. It is estimated that American entrepreneurs invested 5 billion dollars on advertising in 1987, while the expenditure was approximately 50 billion in 1990. The investment reached 15 billion dollars in 2000, whereas the recent investment rocketed to 50 billion dollars [5] same amount it two different years—one must be wrong. In a low-carbon supply chain, promotion is indispensable to ensure low-carbon products receive maximum diffusion. In a closed-loop supply chain, the investment in promotion influences not only the pricing strategy of a forward supply chain, but also the channel recycling strategy of a reverse supply chain. Retailers can promote low-carbon products by explaining their benefits, recommending them to customers and setting up prominent display showcases, etc., which can effectively guide customers towards a low-carbon consumption mindset, and encourage the transformation of consumers from non-environmental friendly to environmental friendly.

Cooperation or competition usually exist within a supply chain or between supply chains. When in the market there exists more than one supply chain, the situation will become complex [6]. The production of manufacturers is usually put into the market by several retailers. Competition will definitely exist among these retailers because they are selling similar products. For instance, home appliance retailers would compete with each other for the market through pricing or non-pricing events; compare with the fierce fight in e-commerce in 2013. Therefore, while considering the situation of retailers’ competition, research on the operational issues of CLSC will be of great significance.

To this end, we consider the low carbon CLSC with a manufacturer who directly collects used-products and reduces carbon emissions as well as two retailers who practice competitive pricing and low carbon promotion. We compare optimal decision results with profits under different cooperation models by systematic comparison and numerical experiments.

The remainder of this paper is organized as follows: Section 2 surveys the related literature while emphasizing the contribution of our work. Section 3 describes the model notations and assumptions in detail. Section 4 presents five different cooperation models. Section 5 provides a comparative analysis of the optimal decision results and profits under the five models. Section 6 provides numerical evidence which confirms the above proposition and supplements some of the analysis in Section 5. In Section 7, some relevant conclusions about the CLSC model are formulated, and, finally, the Appendix contains the proofs of the mathematical statements in the paper.

## 2. Literature Review

The literature closely related to our work can be classified into three broad categories: the decision process of low-carbon supply chains and green supply chains, decision-making and cooperation in supply chains considering promotion strategy, the members’ competition in supply chains. First, our paper is related to the literature on the decision-making process of low-carbon (green) supply chains. Some researchers have studied different coordinating mechanisms in the process of carbon emission reduction by manufacturers. Ghosh and Shah [7] studied the impact of greening costs and consumer sensitivity towards green apparel, and proposed a two-part tariff contract to coordinate the green channel. The same authors also explored supply chain coordination issues arising out of green supply chain initiatives and the impact of cost sharing contract on the key decisions of supply chain players undertaking green initiatives [8]. Government intervention is included in the study of low carbon supply chain. Benjaafar et al. [9] analyzed the impact of carbon caps, carbon trading, carbon taxes and supply chain members’ cooperation on carbon emissions. Xu et al. [10] studied the joint production and pricing problem of a manufacturing firm with multiple products under cap-and-trade and carbon tax regulations, and compared the effects of the two regulations on the total carbon emissions, the firm’s profits and social welfare. Yang and Xiao [6] developed three game models of a green supply chain with governmental interventions under fuzzy uncertainties of both manufacturing cost and consumer demand, and studied how prices, green levels and expected profits are influenced by channel leadership and governmental interventions. The competition of the substitute products and sales channels is considered in the low-carbon supply chain. Zhang et al. [11] investigated the pricing strategies of a green supply chain in which the manufacturer made green products and non-green products together. Zhang et al. [12] focused on the impact of consumer environmental awareness on order quantities and channel coordination within a model with one-manufacturer who produces two types of products and with one-retailer supply chain. Li et al. [13] examined a dual-channel supply chain in which the manufacturer makes green products for environmentally conscious customers. Advertising strategy is considered in the low-carbon supply chain. Ji et al. [14] focused on the carbon emission reduction behaviors for the chain members in both the retail-channel and dual-channel cases using the Stackelberg game model, and analyzed a detailed model which incorporates both cap-and-trade regulation and consumers’ low-carbon preference. Zhou et al. [15] analyzed how the co-op promotion contract and the co-op advertising and emission reduction cost sharing contracts impact the low-carbon supply chain’s optimal decision and coordination. Previous research also proves the impact of carbon emission reduction/green level decisions in forward logistics.

The second stream of research related to our work involves the decision-making process and cooperation in a supply chain with a promotion strategy. Some topics focus on cooperative advertising in a manufacturer-retailer supply scenario. In this article, the main difference is the structure of the price demand and the advertising demand. Xie et al. [16] considering the impact of demand on price and advertising costs of a two echelon supply chain, obtained the retailers’ optimal ordering strategy and suppliers’ buy-back strategy based on the analysis and solution of the supply chain. Miret et al. [5] considered vertical co-op advertising along with pricing decisions in a supply chain, and established four game-theoretic models to study the effect of supply chain power balance on the optimal decisions of supply chain members. Aust and Buscher [17] expanded the existing research which deals with advertising and pricing decisions in a manufacturer–retailer supply chain contemporaneously on the basis of previous studies. Chaab and Rasti-Barzoki [18] mainly analyzed how the advertising factors of the manufacturer and retailer affected the optimal decision results and profits. Advertising strategy is considered in the CLSC. Hong et al. [19] found that cooperative advertising cannot coordinate the CLSC, but a simple two-part tariff contract can coordinate the members of the decentralized CLSC by generating the same performance as in a centralized decision-making system. Gao et al. [20] considering the demand expansion effectiveness of collection effort and sales effort, explored the influence of different channel power structures on the optimal decisions and performance of CLSC. Advertising strategy is also considered in the low-carbon supply chain (Ji et al. [14]; Zhou et al. [15]). Advertising strategy appears in other studies too. Song et al. [21] studied integrated firms’ innovation and advertising decisions in a two-echelon supply chain. Martín-Herrán and Sigué [22] in a bilateral monopoly, investigated the optimal scheduling of retailer and manufacturer advertising in a three-period planning horizon. The above-mentioned studies only consider the advertising strategy of the supply chain with a manufacturer and a retailer, but they did not consider the competition between two retailers’.

Third, competition in and between supply chains has frequently been the subject of research in the past. Some authors consider only price competition. Xiao and Qin [23] studied the coordination of a supply chain with one manufacturer and two competing retailers after the production cost of the manufacturer was disrupted. Jena and Sarmah [24] studied co-operation and competition issues in a closed-loop supply chain with two manufacturers who are competitively selling their new product as well as collecting the used-products for remanufacturing through a common retailer. Ma et al. [25] considered a three-echelon closed-loop supply chain consisting of a single manufacturer, a single retailer and two recyclers and focused on how cooperative strategies affect closed-loop supply chain decision-making. The green supply chain has received increased attention in recent years, thus some researchers begin to consider price competition as well as competition in carbon emission level reduction. Zhu and He [26] investigated green product design issues in supply chains under competition. Liu et al. [27] studied the influence of environmental awareness and competition on the profit of supply chain members. Yang et al. [28] considered two competitive supply chains under the cap-and-trade scheme, each of which consists of one manufacturer and one retailer, and solved and compared the equilibrium solutions of supply chains with several different structures. Wei et al. [29] explored the optimal pricing and warranty period strategies for two complementary products in a supply chain with two manufacturers and one common retailer from a two-stage game theoretic perspective. A few scholars have studied the impact of the price and service competition on supply chain decisions. Wu [30] considered a supply chain consisting of two manufacturers who bundle their products with services and a retailer, and identified the equilibrium characteristics with respect to the remanufacturer’s effort and price and service decisions for all members of the supply chain. Cooperation is common in competition. The above literatures only consider the vertical competition of members in the supply chain, but except [28], they don’t consider the horizontal cooperation.

The main results and contributions of this paper may be summarized as follows: (i) we consider that two retailers set competitive pricing and low carbon promotion in the low-carbon CLSC; (ii) we consider that a vertical cooperation strategy of the manufacturer and retailers is implemented by the manufacturer by providing a subsidy for low-carbon product promotion to the retailers, and the horizontal cooperation strategy of the retailers is implemented by the retailers’ joint decision making; (iii) we suggest how the retailers should choose a cooperation strategy in a competitive environment.

## 3. Notations and Hypothesis

**Figure 1:**
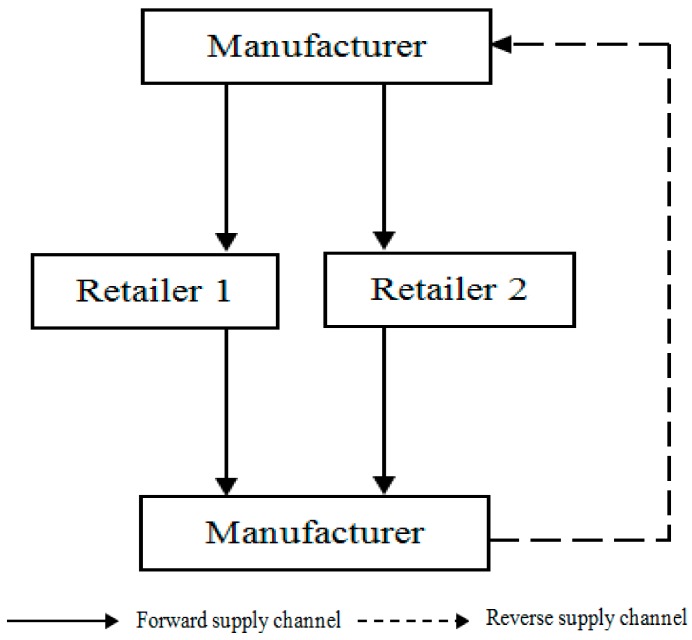
Closed-loop supply chain structure.

In this study, we consider a CLSC with a manufacturer and two retailers, where the manufacturer produces end products from used-products and new materials. The manufacturer is responsible for the carbon emission reduction in this process, returns of used-products from its channels and also sells its products to two retailers. The two retailers competitively promote the end products and sell them to the consumers (as shown in Figure 1). The members in the CLSC make decisions based on their profit maximization. To establish the model, some assumptions are provided as follows:

**Hypothesis** **1.** We assume that remanufactured products cannot satisfy all demand, thus the manufacturer also needs to produce some new products. Both types of product have the same function and they are sold together by the retailer in the same market. The Stackelberg game occurs between the manufacturer and other agents, where the manufacturer behaves as the Stackelberg leader. Moreover, the information is symmetric.

**Hypothesis** **2.** *Chitra has pointed out that consumer awareness of environmental protection has become an important factor affecting their willingness to pay [31]. When the consumers have stronger environmental protection preferences, they will pay a higher price for environmentally-friendly products. Johnson thought that consumers know the product or service of an enterprise when they receive its promotional advertisements [32]. In retailing, the retailer can positively influence the market demand by exerting promotional efforts. When the two retailers compete on the retail price and low-carbon promotion effort, the market demand is a linear function of the retail price $p_{x}$ and $p_{y}$, low-carbon promotion effort $\upsilon_{x}$ and $\upsilon_{y}$ and carbon emission reduction level e, i.e.,* $d_{x}=a-p_{x}+\theta p_{y}+\gamma \left(\upsilon_{x}-\theta \upsilon_{y}\right)+\lambda e$*,* x=1,2, y=3−x*, s.t.* a>0*,* $p_{x}>0$*. Greater* θ *indicates that retailer* x *and retailer* y *is more competitive, and we derive* 0<θ<1 *(e.g., Song et al. [21]; Liu et al. [27]; Zhu and He [26]; Wu [30]).*

**Hypothesis** **3.** *The CLSC decisions are considered in a single-period setting. We assume that some of the products sold in previous periods can be recycled. The unit cost of producing end products from used-products is lower than that from new materials, i.e.,* $c_{r}<c_{m}$ *, let* $\delta =c_{m}-c_{r}>0$ *, where* δ *is the unit cost savings of remanufacturing* *. The average unit cost is* $\bar{c}=\left(1-\tau \right)c_{m}+\tau c_{r}=c_{m}-\delta \tau$ *.*

**Hypothesis** **4.** *The collection rate is usually modeled as a function of the investment in used product collection [1]. In this paper, the collection rate reasonably depends on the investments of manufacturer in recycling activities. More specifically, the collection rate is reasonably formulated as a monotonic increasing function of its own investment. In this paper the collection rate of the manufacturer is formulated as follow:* $\tau =\sqrt{\frac{I}{C_{L}}}$ *, where* I *, whose formulations is given as follow:* $I=C_{L}\tau^{2}$ *, denotes the effective investment of the manufacturer in recycling activities.*

**Hypothesis** **5.** *To reduce carbon emissions, the manufacturer must invest in employing low-carbon technologies. We assume that the manufacturer will induce a total cost during reducing carbon emissions on production period, which is an increasing and convex function of carbon emission reduction level* e *and defined as* $C\left(e\right)=\frac{1}{2}Ue^{2}$ *, we assume that* e>0 *(e.g., Moltó et al. [33]; Zhou et al. [15]; Wei et al. [29]).*

**Hypothesis** **6.** *With an increase in low-carbon promotion cost, the promotion efforts will be decelerated with an increasing impact (e.g., Zhang et al. [34]; Zhou et al. [15]; Song et al. [21]; Ji et al. [14]). We have also assumed that two retailers’ competition is symmetric, thus we consider the promotion costs of retailer as* $C\left(\upsilon_{x}\right)=\frac{1}{2}K{\left(\upsilon_{x}\right)}^{2}$.

The notations defined in Table 1 are used in the model.

Superscript o∈{N,MR,RR,MRR,C} denotes respectively the completely decentralized model (N), M-R vertical cooperation model (MR), R-R horizontal cooperation model (RR), M-R-R vertical and horizontal cooperation model (MRR) and completely centralized model (C). Subscript $f\in \left\{M,R_{x},T\right\}$ denotes respectively the manufacturer, the retailers x and the entire supply chain.

## 4. Model Formulation and Analysis

**Figure 2:**
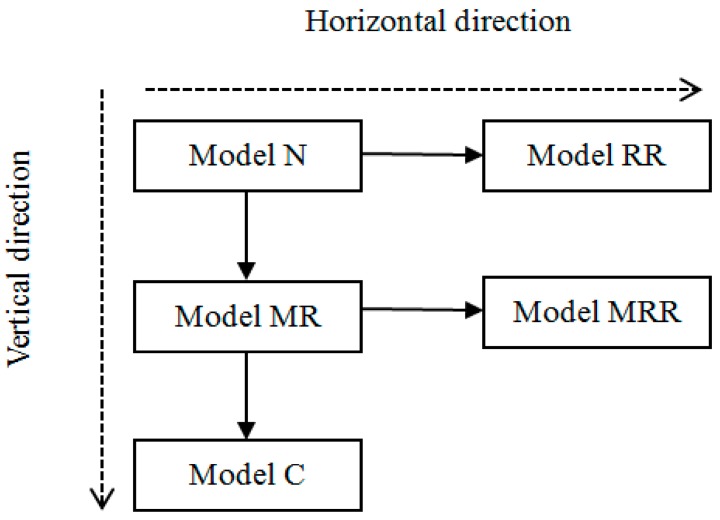
Organization order of the models.

This section primarily analyzes vertical and horizontal cooperation models in the low-carbon CLSC, viz., Model N, Model MR, Model RR, Model MRR and Model C, and their effect on members’ optimal decisions and profits (as shown in Figure 2). We solve these models to obtain optimal decisions and compare the optimal decision variables such as the wholesale price, the retail price, the carbon emission level, the low-carbon promotion effort, the used-products return rate, and the profits of the manufacturer, the retailer and the entire supply chain.

### 4.1. Completely Decentralized Model (N Model)

In this model, the manufacturer and the retailers make their decisions independently to maximize their own profit. The manufacturer decides the wholesale price, the carbon emission level and the used-products return rate. Two retailers independently decide their own retail prices and low-carbon promotion efforts. Thus, we can formulate the decision problem of the manufacturer by:

(1) $$\underset{w,\tau ,e}{\max}\;\pi_{M}^{N}=\left(w-c_{m}+\delta \tau \right)\left(d_{1}+d_{2}\right)-\frac{1}{2}C_{L}\tau^{2}-\frac{1}{2}Ue^{2}$$

as well as the decision problem of the retailers:

(2) $$\underset{p_{x},\upsilon_{x}}{\max}\;\pi_{R_{x}}^{N}=\left(p_{x}-w\right)d_{x}-\frac{1}{2}K{\left(\upsilon_{x}\right)}^{2}$$

From Equations (1) and (2), we have the following results (see the Appendix A.1 for the proof).

**Theorem** **1.** *In the N Model, the optimal decisions of the manufacturer and the retailers are:*

$$w^{N*}=\frac{\left\{aU\left[K\left(2-\theta \right)-\gamma^{2}\left(1-\theta \right)\right]+\left[KU\left(2-3\theta +\theta^{2}\right)-2K\lambda^{2}-U\gamma^{2}{\left(1-\theta \right)}^{2}\right]c_{m}\right\}C_{L}-2aKU\delta^{2}\left(1-\theta \right)}{2\left\{\left[KU\left(2-3\theta +\theta^{2}\right)-K\lambda^{2}-U\gamma^{2}{\left(1-\theta \right)}^{2}\right]C_{L}-KU\delta^{2}{\left(1-\theta \right)}^{2}\right\}}$$

$$\tau^{N*}=\frac{KU\delta \left(1-\theta \right)\left[a-\left(1-\theta \right)c_{m}\right]}{\left[KU\left(2-3\theta +\theta^{2}\right)-K\lambda^{2}-U\gamma^{2}{\left(1-\theta \right)}^{2}\right]C_{L}-KU\delta^{2}{\left(1-\theta \right)}^{2}}$$

$$e^{N*}=\frac{K\lambda \left[a-\left(1-\theta \right)c_{m}\right]C_{L}}{\left[KU\left(2-3\theta +\theta^{2}\right)-K\lambda^{2}-U\gamma^{2}{\left(1-\theta \right)}^{2}\right]C_{L}-KU\delta^{2}{\left(1-\theta \right)}^{2}}$$

$$p_{x}^{N*}=\frac{\left\{aU\left[K\left(3-2\theta \right)-\gamma^{2}\left(1-\theta \right)\right]+\left[KU\left(1-\theta \right)-2K\lambda^{2}-U\gamma^{2}{\left(1-\theta \right)}^{2}\right]c_{m}\right\}C_{L}-2aKU\delta^{2}\left(1-\theta \right)}{2\left\{\left[KU\left(2-3\theta +\theta^{2}\right)-K\lambda^{2}-U\gamma^{2}{\left(1-\theta \right)}^{2}\right]C_{L}-KU\delta^{2}{\left(1-\theta \right)}^{2}\right\}}$$

$$\upsilon_{x}^{N*}=\frac{U\gamma \left(1-\theta \right)\left[a-\left(1-\theta \right)c_{m}\right]C_{L}}{2\left\{\left[KU\left(2-3\theta +\theta^{2}\right)-K\lambda^{2}-U\gamma^{2}{\left(1-\theta \right)}^{2}\right]C_{L}-KU\delta^{2}{\left(1-\theta \right)}^{2}\right\}}$$

By the definition of $d_{x}$, we get the optimal market demand in the N Model:

$$d^{N*}=\frac{KU\left(1-\theta \right)\left[a-\left(1-\theta \right)c_{m}\right]C_{L}}{\left[KU\left(2-3\theta +\theta^{2}\right)-K\lambda^{2}-U\gamma^{2}{\left(1-\theta \right)}^{2}\right]C_{L}-KU\delta^{2}{\left(1-\theta \right)}^{2}}$$

Substituting the value of $w^{N*}$, $\tau^{N*}$, $e^{N*}$, $p_{x}^{N*}$ and $\upsilon_{x}^{N*}$ into Equations (1) and (2), the profits of the manufacturer, the retailers and the entire supply chain are given by $\pi_{T}^{MR*}=\pi_{M}^{MR*}+\pi_{R}^{MR*}$:

$$\pi_{M}^{N*}=\frac{KU{\left[a-\left(1-\theta \right)c_{m}\right]}^{2}C_{L}}{2\left\{\left[KU\left(2-3\theta +\theta^{2}\right)-K\lambda^{2}-U\gamma^{2}{\left(1-\theta \right)}^{2}\right]C_{L}-KU\delta^{2}{\left(1-\theta \right)}^{2}\right\}}$$

$$\pi_{R_{x}}^{N*}=\frac{KU^{2}\left(2K-\gamma^{2}\right){\left(1-\theta \right)}^{2}{\left[a-\left(1-\theta \right)c_{m}\right]}^{2}C_{L}^{2}}{8{\left\{\left[KU\left(2-3\theta +\theta^{2}\right)-K\lambda^{2}-U\gamma^{2}{\left(1-\theta \right)}^{2}\right]C_{L}-KU\delta^{2}{\left(1-\theta \right)}^{2}\right\}}^{2}}$$

$$\pi_{T}^{N*}=\frac{KU{\left[a-\left(1-\theta \right)c_{m}\right]}^{2}C_{L}\left\{\left[2KU\left(3-5\theta +2\theta^{2}\right)-2K\lambda^{2}-3U\gamma^{2}{\left(1-\theta \right)}^{2}\right]C_{L}-2KU\delta^{2}{\left(1-\theta \right)}^{2}\right\}}{4{\left\{\left[KU\left(2-3\theta +\theta^{2}\right)-K\lambda^{2}-U\gamma^{2}{\left(1-\theta \right)}^{2}\right]C_{L}-KU\delta^{2}{\left(1-\theta \right)}^{2}\right\}}^{2}}$$

The result in Theorem 1 shows that two retailers’ optimal decision results and profits are the same. This means that when two retailers make the same decision, their equilibrium solutions will be identical because of our assumptions of symmetry of the parameters between the two market demands. The optimal solutions are all related to the market parameters and cost parameters, which the optimal solutions generally increase in market parameters of γ or λ. The N Model provides a benchmark scenario to compare with other models. A complete proof can be found in Appendix B.

### 4.2. M-R Vertical Cooperation Model (MR Model)

In the MR Model, the manufacturer can cooperate with retailers by a contract whereby the manufacturer provides low-carbon product promotion subsidies to the retailers. The members’ decision making is similar to the N Model. Therefore, we can formulate the decision problem of the manufacturer as:

(3) $$\underset{w,\tau ,e}{\max\;}\pi_{M}^{MR}=\left(w-c_{m}+\delta \tau -\xi \upsilon_{1}\right)d_{1}+\left(w-c_{m}+\delta \tau -\xi \upsilon_{2}\right)d_{2}-\frac{1}{2}C_{L}\tau^{2}-\frac{1}{2}Ue^{2}$$

as well as the decision problem of the retailers:

(4) $$\underset{p_{x},\upsilon_{x}}{\max}\;\pi_{R_{x}}^{MR}=\left(p_{x}-w+\xi \upsilon_{x}\right)d_{x}-\frac{1}{2}K{\left(\upsilon_{x}\right)}^{2}$$

From Equations (3) and (4), we have the following results (see the Appendix A.2 for the proof).

**Theorem** **2.** *In the MR Model, the optimal decisions of the manufacturer and the retailers are:*

$$w^{MR*}=\frac{\{aU\left[K\left(2-\theta \right)-\left(1-\theta \right)\left(\gamma^{2}-\xi^{2}\right)\right]+\left[KU\left(2-3\theta +\theta^{2}\right)-2K\lambda^{2}-U{\left(1-\theta \right)}^{2}{\left(\gamma +\xi \right)}^{2}\right]c_{m}\}C_{L}-2aKU\delta^{2}\left(1-\theta \right)}{2\left\{\left[KU\left(2-3\theta +\theta^{2}\right)-K\lambda^{2}-U\gamma {\left(1-\theta \right)}^{2}\left(\gamma +\xi \right)\right]C_{L}-KU\delta^{2}{\left(1-\theta \right)}^{2}\right\}},$$

$$\tau^{MR*}=\frac{KU\delta \left(1-\theta \right)\left[a-\left(1-\theta \right)c_{m}\right]}{\left[KU\left(2-3\theta +\theta^{2}\right)-K\lambda^{2}-U\gamma {\left(1-\theta \right)}^{2}\left(\gamma +\xi \right)\right]C_{L}-KU\delta^{2}{\left(1-\theta \right)}^{2}}$$

$$e^{MR*}=\frac{K\lambda \left[a-\left(1-\theta \right)c_{m}\right]C_{L}}{\left[KU\left(2-3\theta +\theta^{2}\right)-K\lambda^{2}-U\gamma {\left(1-\theta \right)}^{2}\left(\gamma +\xi \right)\right]C_{L}-KU\delta^{2}{\left(1-\theta \right)}^{2}}$$

$$p_{x}^{MR*}=\frac{\left\{aU\left[K\left(3-2\theta \right)-\gamma \left(1-\theta \right)\left(\gamma +\xi \right)\right]+\left[KU\left(1-\theta \right)-2K\lambda^{2}-U\gamma {\left(1-\theta \right)}^{2}\left(\gamma +\xi \right)\right]c_{m}\right\}C_{L}-2aKU\delta^{2}\left(1-\theta \right)}{2\left\{\left[KU\left(2-3\theta +\theta^{2}\right)-K\lambda^{2}-U\gamma {\left(1-\theta \right)}^{2}\left(\gamma +\xi \right)\right]C_{L}-KU\delta^{2}{\left(1-\theta \right)}^{2}\right\}}$$

$$\upsilon_{x}^{MR*}=\frac{U\left(1-\theta \right)\left(\gamma +\xi \right)\left[a-\left(1-\theta \right)c_{m}\right]C_{L}}{2\left\{\left[KU\left(2-3\theta +\theta^{2}\right)-K\lambda^{2}-U\gamma {\left(1-\theta \right)}^{2}\left(\gamma +\xi \right)\right]C_{L}-KU\delta^{2}{\left(1-\theta \right)}^{2}\right\}}$$

By the definition of $d_{x}$, we get the optimal market demand in the MR Model:

$$d^{MR*}=\frac{KU\left(1-\theta \right)\left[a-\left(1-\theta \right)c_{m}\right]C_{L}}{\left[KU\left(2-3\theta +\theta^{2}\right)-K\lambda^{2}-U\gamma {\left(1-\theta \right)}^{2}\left(\gamma +\xi \right)\right]C_{L}-KU\delta^{2}{\left(1-\theta \right)}^{2}}$$

Substituting the value of $w^{MR*}$, $\tau^{MR*}$, $e^{MR*}$, $p_{x}^{MR*}$ and $\upsilon_{x}^{MR*}$ into Equations (3) and (4), the profits of the manufacturer, the retailers and the entire supply chain are given by $\pi_{T}^{MR*}=\pi_{M}^{MR*}+\pi_{R}^{MR*}:$

$$\pi_{M}^{MR*}=\frac{KU{\left[a-\left(1-\theta \right)c_{m}\right]}^{2}C_{L}}{2\left\{\left[KU\left(2-3\theta +\theta^{2}\right)-K\lambda^{2}-U\gamma {\left(1-\theta \right)}^{2}\left(\gamma +\xi \right)\right]C_{L}-KU\delta^{2}{\left(1-\theta \right)}^{2}\right\}}$$

$$\;\pi_{R_{x}}^{MR*}=\frac{KU^{2}{\left(1-\theta \right)}^{2}\left[2K-{\left(\gamma +\xi \right)}^{2}\right]{\left[a-\left(1-\theta \right)c_{m}\right]}^{2}C_{L}^{2}}{8{\left\{\left[KU\left(2-3\theta +\theta^{2}\right)-K\lambda^{2}-U\gamma {\left(1-\theta \right)}^{2}\left(\gamma +\xi \right)\right]C_{L}-KU\delta^{2}{\left(1-\theta \right)}^{2}\right\}}^{2}}$$

$$\pi_{T}^{MR*}=\frac{KU{\left[a-\left(1-\theta \right)c_{m}\right]}^{2}C_{L}\left\{\left[2KU\left(3-5\theta +2\theta^{2}\right)-2K\lambda^{2}-U{\left(1-\theta \right)}^{2}\left(3\gamma^{2}+4\gamma \xi +\xi^{2}\right)\right]C_{L}-2KU\delta^{2}{\left(1-\theta \right)}^{2}\right\}}{4{\left\{\left[KU\left(2-3\theta +\theta^{2}\right)-K\lambda^{2}-U\gamma {\left(1-\theta \right)}^{2}\left(\gamma +\xi \right)\right]C_{L}-KU\delta^{2}{\left(1-\theta \right)}^{2}\right\}}^{2}}$$

The result in Theorem 2 notes that the optimal carbon emissions reduction level, return rate, low-carbon promotion effort, market demand and manufacturer’s profit increases with the subsidy rate. This means that when the manufacturer increases the amount of the low-carbon product promotion subsidy to the retailers, it is conducive to resource recycling, environmental protection and an increase of the manufacturer’s profit, but by comparing with the N Model, the retailers gladly accept the subsidies from the manufacturer when $0<\xi \leq \xi_{1}$ and $0<\theta \leq \theta_{0}$.

Here:

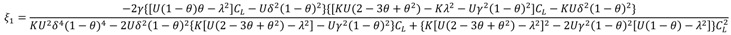

$$\theta_{0}=\frac{\left(2\delta^{2}+C_{L}\right)\sqrt{U}-\sqrt{C_{L}}\sqrt{\left(U-4\lambda^{2}\right)C_{L}-4\delta^{2}\lambda^{2}}}{2\sqrt{U}\left(\delta^{2}+C_{L}\right)}$$

### 4.3. R-R Horizontal Cooperation Model (RR Model)

In the RR Model, the two retailers can cooperate to act in unison in order to maximize their total profits. The manufacturer decides the wholesale price, the carbon emission level and the used-products return rate. Two retailers jointly decide the retail price and the low-carbon promotion efforts Thus, we can formulate the decision problem of the manufacturer:

(5) $$\underset{w,\tau ,e}{\max}\;\pi_{M}^{RR}=\left(w-c_{m}+\delta \tau \right)\left(d_{1}+d_{2}\right)-\frac{1}{2}C_{L}\tau^{2}-\frac{1}{2}Ue^{2}$$

as well as the decision problem of the retailers’ horizontal cooperation:

(6) $$\underset{p_{x},\upsilon_{x}}{\max}\;\pi_{R}^{RR}=\left(p_{1}-w\right)d_{1}+\left(p_{2}-w\right)d_{2}-\frac{1}{2}K{\left(\upsilon_{1}\right)}^{2}-\frac{1}{2}K{\left(\upsilon_{2}\right)}^{2}$$

where $\pi_{R}^{RR}=\pi_{R_{1}}+\pi_{R_{2}}$

From Equations (5) and (6), we have the following results (see the Appendix A.3 for the proof).

**Theorem** **3.** *In the RR Model, the optimal decisions of the manufacturer and the retailers are:*

$$w^{RR*}=\frac{\left\{aU\left[2K-\gamma^{2}\left(1-\theta \right)\right]+\left[2KU\left(1-\theta \right)-2K\lambda^{2}-U\gamma^{2}{\left(1-\theta \right)}^{2}\right]c_{m}\right\}C_{L}-2aKU\delta^{2}\left(1-\theta \right)}{2\left\{\left[2KU\left(1-\theta \right)-K\lambda^{2}-U\gamma^{2}{\left(1-\theta \right)}^{2}\right]C_{L}-KU\delta^{2}{\left(1-\theta \right)}^{2}\right\}}$$

$$\tau^{RR*}=\frac{KU\delta \left(1-\theta \right)\left[a-\left(1-\theta \right)c_{m}\right]}{\left[2KU\left(1-\theta \right)-K\lambda^{2}-U\gamma^{2}{\left(1-\theta \right)}^{2}\right]C_{L}-KU\delta^{2}{\left(1-\theta \right)}^{2}}$$

$$e^{RR*}=\frac{K\lambda \left[a-\left(1-\theta \right)c_{m}\right]C_{L}}{\left[2KU\left(1-\theta \right)-K\lambda^{2}-U\gamma^{2}{\left(1-\theta \right)}^{2}\right]C_{L}-KU\delta^{2}{\left(1-\theta \right)}^{2}}$$

$$p_{x}^{RR*}=\frac{\left\{aU\left[3K-\gamma^{2}\left(1-\theta \right)\right]+\left[KU\left(1-\theta \right)-2K\lambda^{2}-U\gamma^{2}{\left(1-\theta \right)}^{2}\right]c_{m}\right\}C_{L}-2aKU\delta^{2}\left(1-\theta \right)}{2\left\{\left[2KU\left(1-\theta \right)-K\lambda^{2}-U\gamma^{2}{\left(1-\theta \right)}^{2}\right]C_{L}-KU\delta^{2}{\left(1-\theta \right)}^{2}\right\}}$$

$$\upsilon_{x}^{RR*}=\frac{U\gamma \left(1-\theta \right)\left[a-\left(1-\theta \right)c_{m}\right]C_{L}}{2\left\{\left[2KU\left(1-\theta \right)-K\lambda^{2}-U\gamma^{2}{\left(1-\theta \right)}^{2}\right]C_{L}-KU\delta^{2}{\left(1-\theta \right)}^{2}\right\}}$$

By the definition of $d_{x}$, we get the optimal market demand in the RR Model:

$$d^{RR*}=\frac{KU\left(1-\theta \right)\left[a-\left(1-\theta \right)c_{m}\right]C_{L}}{\left[2KU\left(1-\theta \right)-K\lambda^{2}-U\gamma^{2}{\left(1-\theta \right)}^{2}\right]C_{L}-KU\delta^{2}{\left(1-\theta \right)}^{2}}$$

Substituting the value of $w^{RR*}$, $\tau^{RR*}$, $e^{RR*}$, $p_{x}^{RR*}$ and $\upsilon_{x}^{RR*}$ into Equations (5) and (6), the profits of the manufacturer, the retailers and the entire supply chain are given by $\pi_{T}^{RR*}=\pi_{M}^{RR*}+\pi_{R}^{RR*}:$

$$\pi_{M}^{RR*}=\frac{KU{\left[a-\left(1-\theta \right)c_{m}\right]}^{2}C_{L}}{2\left\{\left[2KU\left(1-\theta \right)-K\lambda^{2}-U\gamma^{2}{\left(1-\theta \right)}^{2}\right]C_{L}-KU\delta^{2}{\left(1-\theta \right)}^{2}\right\}}$$

$$\pi_{R_{x}}^{RR*}=\frac{KU^{2}\left[2K-\gamma^{2}\left(1-\theta \right)\right]\left(1-\theta \right){\left[a-\left(1-\theta \right)c_{m}\right]}^{2}C_{L}^{2}}{8{\left\{\left[2KU\left(1-\theta \right)-K\lambda^{2}-U\gamma^{2}{\left(1-\theta \right)}^{2}\right]C_{L}-KU\delta^{2}{\left(1-\theta \right)}^{2}\right\}}^{2}}$$

$$\pi_{T}^{RR*}=\frac{KU{\left[a-\left(1-\theta \right)c_{m}\right]}^{2}C_{L}\left\{\left[6KU\left(1-\theta \right)-2K\lambda^{2}-3U\gamma^{2}{\left(1-\theta \right)}^{2}\right]C_{L}-2KU\delta^{2}{\left(1-\theta \right)}^{2}\right\}}{4{\left\{\left[2KU\left(1-\theta \right)-K\lambda^{2}-U\gamma^{2}{\left(1-\theta \right)}^{2}\right]C_{L}-KU\delta^{2}{\left(1-\theta \right)}^{2}\right\}}^{2}}$$

The result in Theorem 3 shows that like the N Model the optimal solutions are all related to the market parameters and cost parameters, which the optimal solutions generally increase in market parameters of γ or λ. A complete proof can be found in the Appendix B.

### 4.4. M-R-R Vertical and Horizontal Cooperation Model (MRR Model)

This model is a combination of the MR Model and the RR Model. Two retailers as a central planner decide the retail price and the low-carbon promotion effort. The manufacturer decides the wholesale price, the carbon emissions reduction level, the low-carbon level and the used-products return rate, and simultaneously provides low-carbon product promotion subsidies to the retailers. Therefore, we can formulate the decision problem of the manufacturer as:

(7) $$\underset{w,\tau ,e}{\max\;}\pi_{M}^{MRR}=\left(w-c_{m}+\delta \tau -\xi \upsilon_{1}\right)d_{1}+\left(w-c_{m}+\delta \tau -\xi \upsilon_{2}\right)d_{2}-\frac{1}{2}C_{L}\tau^{2}-\frac{1}{2}Ue^{2}$$

as well as the decision problem of the retailers’ horizontal cooperation:

(8) $$\underset{p_{x},\upsilon_{x}}{\max\;}\pi_{R}^{MRR}=\left(p_{1}-w+\xi \upsilon_{1}\right)d_{1}+\left(p_{2}-w+\xi \upsilon_{2}\right)d_{2}-\frac{1}{2}K{\left(\upsilon_{2}\right)}^{2}-\frac{1}{2}K{\left(\upsilon_{1}\right)}^{2}$$

where $\pi_{R}^{MRR}=\pi_{R_{1}}+\pi_{R_{2}}$

From Equations (7) and (8), we have the following results (see the Appendix A.4 for the proof).

**Theorem** **4.** *In the MRR Model, the optimal decisions of the manufacturer and the retailers are:*

$$w^{MRR*}=\frac{\left\{aU\left[2K-\left(1-\theta \right)\left(\gamma^{2}-\xi^{2}\right)\right]+\left[2KU\left(1-\theta \right)-2K\lambda^{2}-U{\left(1-\theta \right)}^{2}{\left(\gamma +\xi \right)}^{2}\right]c_{m}\right\}C_{L}-2aKU\delta^{2}\left(1-\theta \right)}{2\left\{\left[2KU\left(1-\theta \right)-K\lambda^{2}-U\gamma {\left(1-\theta \right)}^{2}\left(\gamma +\xi \right)\right]C_{L}-KU\delta^{2}{\left(1-\theta \right)}^{2}\right\}}$$

$$\tau^{MRR*}=\frac{KU\delta \left(1-\theta \right)\left[a-\left(1-\theta \right)c_{m}\right]}{\left[2KU\left(1-\theta \right)-K\lambda^{2}-U\gamma {\left(1-\theta \right)}^{2}\left(\gamma +\xi \right)\right]C_{L}-KU\delta^{2}{\left(1-\theta \right)}^{2}}$$

$$e^{MRR*}=\frac{K\lambda \left[a-\left(1-\theta \right)c_{m}\right]C_{L}}{\left[2KU\left(1-\theta \right)-K\lambda^{2}-U\gamma {\left(1-\theta \right)}^{2}\left(\gamma +\xi \right)\right]C_{L}-KU\delta^{2}{\left(1-\theta \right)}^{2}}$$

$$p_{x}^{MRR*}=\frac{\left\{aU\left[3K-\gamma \left(1-\theta \right)\left(\gamma +\xi \right)\right]+\left[KU\left(1-\theta \right)-2K\lambda^{2}-U\gamma {\left(1-\theta \right)}^{2}\left(\gamma +\xi \right)\right]c_{m}\right\}C_{L}-2aKU\delta^{2}\left(1-\theta \right)}{2\left\{\left[2KU\left(1-\theta \right)-K\lambda^{2}-U\gamma {\left(1-\theta \right)}^{2}\left(\gamma +\xi \right)\right]C_{L}-KU\delta^{2}{\left(1-\theta \right)}^{2}\right\}}$$

$$\upsilon_{x}^{MRR*}=\frac{U\left(1-\theta \right)\left(\gamma +\xi \right)\left[a-\left(1-\theta \right)c_{m}\right]C_{L}}{2\left\{\left[2KU\left(1-\theta \right)-K\lambda^{2}-U\gamma {\left(1-\theta \right)}^{2}\left(\gamma +\xi \right)\right]C_{L}-KU\delta^{2}{\left(1-\theta \right)}^{2}\right\}}$$

By the definition of $d_{x}$, we get the optimal market demand in the MRR Model:

$$d^{MRR*}=\frac{KU\left(1-\theta \right)\left[a-\left(1-\theta \right)c_{m}\right]C_{L}}{\left[2KU\left(1-\theta \right)-K\lambda^{2}-U\gamma {\left(1-\theta \right)}^{2}\left(\gamma +\xi \right)\right]C_{L}-KU\delta^{2}{\left(1-\theta \right)}^{2}}$$

Substituting the values of $w^{MRR*}$, $\;\tau^{MRR*}$, $e^{MRR*}$, $p_{x}^{MRR*}$ and $\upsilon_{x}^{MRR*}$ into Equations (7) and (8), the profits of the manufacturer, the retailers and the entire supply chain are given by $\pi_{T}^{MRR*}=\pi_{M}^{MRR*}+\pi_{R}^{MRR*}$:

$$\pi_{M}^{MRR*}=\frac{KU{\left[a-\left(1-\theta \right)c_{m}\right]}^{2}C_{L}}{2\left\{\left[2KU\left(1-\theta \right)-K\lambda^{2}-U\gamma {\left(1-\theta \right)}^{2}\left(\gamma +\xi \right)\right]C_{L}-KU\delta^{2}{\left(1-\theta \right)}^{2}\right\}}$$

$$\pi_{R_{x}}^{MRR*}=\frac{KU^{2}\left(1-\theta \right)\left[2K-\left(1-\theta \right){\left(\gamma +\xi \right)}^{2}\right]{\left[a-\left(1-\theta \right)c_{m}\right]}^{2}C_{L}^{2}}{8{\left\{\left[2KU\left(1-\theta \right)-K\lambda^{2}-U\gamma {\left(1-\theta \right)}^{2}\left(\gamma +\xi \right)\right]C_{L}-KU\delta^{2}{\left(1-\theta \right)}^{2}\right\}}^{2}}$$

$$\pi_{T}^{MRR*}=\frac{KU{\left[a-\left(1-\theta \right)c_{m}\right]}^{2}C_{L}\left\{\left[6KU\left(1-\theta \right)-2K\lambda^{2}-U{\left(1-\theta \right)}^{2}\left(3\gamma^{2}+4\gamma \xi +\xi^{2}\right)\right]C_{L}-2KU\delta^{2}{\left(1-\theta \right)}^{2}\right\}}{4{\left\{\left[2KU\left(1-\theta \right)-K\lambda^{2}-U\gamma {\left(1-\theta \right)}^{2}\left(\gamma +\xi \right)\right]C_{L}-KU\delta^{2}{\left(1-\theta \right)}^{2}\right\}}^{2}}$$

where:

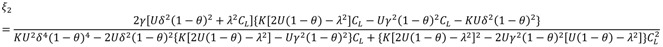


Like the MR Model, when the manufacturer increases the intensity of the low-carbon promotion subsidies for the retailers, it is conducive to resource recycling, environmental protection and increased manufacturer’s profits. Different from the MR Model, this model is compared with the RR Model. The retailers gladly accept the subsidies from the manufacturer when $0<\xi \leq \xi_{2}$, and the retailer needs more global awareness to accept the manufacturers’ cooperation if $\xi_{2}<\xi <\gamma$. A complete proof can be found in the Appendix B.

### 4.5. Completely Centralized Model (C Model)

In this model, all members as an integrated system decide the retailer price, the carbon emissions reduction level, the low-carbon promotion and the used-products return rate to maximize the profit of the entire supply chain. Therefore, we can formulate the decision problem of the entire supply chain:

(9) $$\underset{p_{x},\upsilon_{x},e,\tau }{\max}\;\pi_{T}^{C}=\left(p_{1}-c_{m}+\delta \tau \right)d_{1}+\left(p_{2}-c_{m}+\delta \tau \right)d_{2}-\frac{1}{2}C_{L}\tau^{2}-\frac{1}{2}Ue^{2}-\frac{1}{2}K{\left(\upsilon_{1}\right)}^{2}-\frac{1}{2}K{\left(\upsilon_{2}\right)}^{2}$$

where $\pi_{T}^{C}=\pi_{M}+\pi_{R_{1}}+\pi_{R_{1}}$.

From Equation (9), we have the following results (see the Appendix A.5 for the proof).

**Theorem** **5.** *In the C Model, the optimal decisions of the entire supply chain are:*

$$\tau^{C*}=\frac{2KU\delta \left(1-\theta \right)\left[a-\left(1-\theta \right)c_{m}\right]}{\left[2KU\left(1-\theta \right)-2K\lambda^{2}-U\gamma^{2}{\left(1-\theta \right)}^{2}\right]C_{L}-2KU\delta^{2}{\left(1-\theta \right)}^{2}}$$

$$e^{C*}=\frac{2K\lambda \left[a-\left(1-\theta \right)c_{m}\right]C_{L}}{\left[2KU\left(1-\theta \right)-2K\lambda^{2}-U\gamma^{2}{\left(1-\theta \right)}^{2}\right]C_{L}-2KU\delta^{2}{\left(1-\theta \right)}^{2}}$$

$$p_{x}^{C*}=\frac{\left\{aKU+\left[KU\left(1-\theta \right)-2K\lambda^{2}-U\gamma^{2}{\left(1-\theta \right)}^{2}\right]c_{m}\right\}C_{L}-2aKU\delta^{2}\left(1-\theta \right)}{\left[2KU\left(1-\theta \right)-2K\lambda^{2}-U\gamma^{2}{\left(1-\theta \right)}^{2}\right]C_{L}-2KU\delta^{2}{\left(1-\theta \right)}^{2}}$$

$$\upsilon_{x}^{C*}=\frac{U\gamma \left(1-\theta \right)\left[a-\left(1-\theta \right)c_{m}\right]C_{L}}{\left[2KU\left(1-\theta \right)-2K\lambda^{2}-U\gamma^{2}{\left(1-\theta \right)}^{2}\right]C_{L}-2KU\delta^{2}{\left(1-\theta \right)}^{2}}$$

By the definition of $d_{x}$, we get the optimal market demand in the C Model:

$$d^{C*}=\frac{2KU\left(1-\theta \right)\left[a-\left(1-\theta \right)c_{m}\right]C_{L}}{\left[2KU\left(1-\theta \right)-2K\lambda^{2}-U\gamma^{2}{\left(1-\theta \right)}^{2}\right]C_{L}-2KU\delta^{2}{\left(1-\theta \right)}^{2}}$$

Substituting the value of $\;\tau^{C*}$, $e^{C*}$, $p_{x}^{C*}$ and $\upsilon_{x}^{C*}$ into Equation (9), the profit of the entire supply chain are given by:

$$\pi_{T}^{C*}=\frac{KU{\left[a-\left(1-\theta \right)c_{m}\right]}^{2}C_{L}}{\left[2KU\left(1-\theta \right)-2K\lambda^{2}-U\gamma^{2}{\left(1-\theta \right)}^{2}\right]C_{L}-2KU\delta^{2}{\left(1-\theta \right)}^{2}}$$

Theorem 5 indicates that the optimal retail price is smallest in the C Model but the optimal return rate, the carbon emissions level, the low-carbon promotion effort and the market demand are best among all models (see Propositions 1–3). A complete proof can be found in the Appendix B.

## 5. Comparative Analysis

In this section, the derived values of the five different models are compared to understand the influence of different models on the optimal decision results and obtain some important management implications in low-carbon CLSC. The comparative results are listed in decreasing order.

**Proposition** **1.** *The ordinal relationship of the manufacturer’s optimal decision results is given by:*

$$\begin{array}{c} w^{MR*}>w^{N*},\;w^{MRR*}>w^{RR*};\;\tau^{C*}>\tau^{MR*}>\tau^{MRR*}>\tau^{N*}>\tau^{RR*},\;e^{C*}>e^{MR*}>e^{MRR*}>e^{N*}>e^{RR*},\;\mathrm{if}\\ 0<\theta <\frac{\gamma \xi }{K+\gamma \xi };\;\tau^{C*}>\tau^{MR*}>\tau^{N*}>\tau^{MRR*}>\tau^{RR*},\;e^{C*}>e^{MR*}>e^{N*}>e^{MRR*}>e^{RR*},\;\mathrm{if}\frac{\gamma \xi }{K+\gamma \xi }<\theta <1. \end{array}$$

Proposition 1 shows that the manufacturer’s optimal decision results would be influenced by various cooperation structures. Moreover, they are determined by the manufacturer. Based on the N Model and RR Model, the vertical cooperation (MR and MRR) increases the wholesale price. In the carbon emissions level and used-product return level, the retailers’ horizontal cooperation (RR) is lowest among all models, and the vertical and horizontal cooperation of the manufacturer and retailers (MRR) increases compared with the RR Model. When the competitive intensity is very small, the vertical and horizontal cooperation of the manufacturer and retailers (MRR) is higher than the no-cooperation (N), but that gradually lower than the no-cooperation (N) as the competition intensity increasing. The vertical cooperation of manufacturer and retailers (MR) is best in all semi cooperation.

**Proposition** **2.** *The ordinal relationship of the retailers’ optimal decision results is given by:*

$$\begin{array}{c} p_{x}^{MRR*}>p_{x}^{RR*}>p_{x}^{C*},\;p_{x}^{MR*}>p_{x}^{N*}>p_{x}^{C*};\;\upsilon_{x}^{C*}>\upsilon_{x}^{MR*}>\upsilon_{x}^{\mathrm{MRR}*}>\upsilon_{x}^{N*}>\upsilon_{x}^{RR*},\;\mathrm{if}\;0<\theta <\frac{2\xi }{\gamma +\xi };\\ \upsilon_{x}^{C*}>\upsilon_{x}^{MR*}>\upsilon_{x}^{N*}>\upsilon_{x}^{\mathrm{MRR}*}>\upsilon_{x}^{RR*},\;\mathrm{if}\;\frac{2\xi }{\gamma +\xi }<\theta <1. \end{array}$$

Proposition 2 shows that the retail prices of the MRR Model and MR Model are individually higher than those of the RR Model and N Model, because the retailers behave as Stackelberg game followers and the manufacturer charges a higher wholesale price in the corresponding models. The low-carbon promotion effort in the RR Model is lowest among all models and that in the MR Model is best in all semi-cooperation. There is a threshold ($\frac{2\xi }{\gamma +\xi }$) of the competition intensity, the ordinal relationship of the market demand will change from the MRR Model to the N Model. This threshold ($\frac{2\xi }{\gamma +\xi }$) is higher than the threshold ($\frac{\gamma \xi }{K+\gamma \xi }$) in Proposition 1.

**Proposition** **3.** *The ordinal relationship of the market demand is given by:*

$$d^{C*}>d^{MR*}>d^{MRR*}>d^{N*}>d^{RR*},\;\mathrm{if}\;0<\theta <\frac{\gamma \xi }{K+\gamma \xi };$$

$$d^{C*}>d^{MR*}>d^{N*}>d^{MRR*}>d^{RR*},\;\mathrm{if}\;\frac{\gamma \xi }{K+\gamma \xi }<\theta <1.$$

Proposition 3 shows that this ordinal relationship of the market demand is affected by the ordinal relationship of the retail price, carbon emissions reduction level and used-product return level. From the above proposition, in returning used products, the MR Model in all semi-cooperation scenarios is the best and the RR Model is the worst. Similarly a threshold ($\frac{\gamma \xi }{K+\gamma \xi }$) of the competition intensity, the returning used products will change from the MRR Model to the N Model.

**Proposition** **4.** *The profits of the manufacturer can be ordered as follows:*

$$\pi_{M}^{MR*}>\pi_{M}^{MRR*}>\pi_{M}^{N*}>\pi_{M}^{RR*},\;\mathrm{if}\;0<\theta <\frac{\gamma \xi }{K+\gamma \xi };\;\pi_{M}^{MR*}>\pi_{M}^{N*}>\pi_{M}^{MRR*}>\pi_{M}^{RR*},\;\mathrm{if}\;\frac{\gamma \xi }{K+\gamma \xi }<\theta <1.$$

Proposition 4 shows that compared with no-cooperation (N), when the competitive intensity is very small, the vertical cooperation of manufacturer and retailers (MR) and vertical and horizontal cooperation of the manufacturer and retailers (MRR) effectively improve the manufacturer’s profits, and the retailers’ horizontal cooperation (RR) reduces the manufacturer’s profits, but only the MR Model effectively improves the manufacturer’s profits, and the MRR Model and RR Model reduce the manufacturer’s profits when the competitive intensity is higher. Proofs of all propositions are provided in Appendix C.

## 6. Numerical Analysis

In this section, we perform a numerical study for the proposed low-carbon CLSC model. Then the impact of the competitive intensity between the two retailers on the decision results and the profits are discussed.

We examine the impact of the competitive intensities θ on the decision results and the profits in the five models. The selected parameter values should satisfy the assumptions mentioned in Section 3, so we assume that the parameters values are: a=50, δ=15, $c_{m}=20$, A=1000, U=300, K=100, λ=5, γ=6 and ξ=1, we take different values of θϵ[0,0.6].

Thus we get results shown in the series of tables below depicting the effect of the competitive intensity on the optimal values in the different models, within the range of values defined above.

Table 2 shows that, with increasing competitive intensity θ, the wholesale prices follow four ordinal relationships: $w^{MRR*}>w^{MR*}>w^{RR*}>w^{N*}$, $w^{MR*}>w^{MRR*}>w^{RR*}>w^{N*}$, $w^{MR*}>w^{MRR*}>w^{N*}>w^{RR*}$ and $w^{MR*}>w^{N*}>w^{MRR*}>w^{RR*}$. That means that all cooperative models improve the wholesale price comparing with no-cooperation model when the value θ is small. The gap between the MR Model and the N Model is bigger than that between the MRR Model and the RR Model. Rate of increase of carbon emissions reduction level and used-products return level in the MR Model and the N Model is faster than those of the MRR Model and the RR Model.

Table 3 shows that competitive intensity changes did not affect the ordinal relationship of the retail price. The retail price increases from the C Model to the MRR Model ($p_{x}^{C*}<p_{x}^{N*}<p_{x}^{MR*}<p_{x}^{RR*}<p_{x}^{MRR*}$). The changing trends of the low-carbon promotion in the RR Model and the MRR Model are slower than those in the N Model and the MR Model.

Table 4 shows that more consumers would like to purchase products when the vertical cooperation of manufacturer and retailers is semi-cooperative. The market demands are effects by the optimal decision results as described above; thus the market demand gap between the RR Model and the MRR Model decreases if competitive intensity of θ increases. Similarly, the changing trends of the market demand in the RR Model and the MRR Model are slower than that in the N Model and the MR Model.

Table 5 show that, from the above proposition, there are four ordinal relationships of the retailers’ profits as $\pi_{Rx}^{MR*}>\pi_{Rx}^{MRR*}>\pi_{Rx}^{N*}>\pi_{Rx}^{RR*}$, $\pi_{Rx}^{MR*}>\pi_{Rx}^{N*}>\pi_{Rx}^{MRR*}>\pi_{Rx}^{RR*}$, $\pi_{Rx}^{N*}>\pi_{Rx}^{MR*}>\pi_{Rx}^{MRR*}>\pi_{Rx}^{RR*}$ and $\pi_{Rx}^{MRR*}>\pi_{Rx}^{RR*}>\pi_{Rx}^{N*}>\pi_{Rx}^{MR*}$ asthe competitive intensity θ increases, because they are affected by the diversity of the ordinal relationship of the wholesale price. In addition, there are two ordinal relationships of retailers’ profits given by $\pi_{T}^{C*}>\pi_{T}^{MR*}>\pi_{T}^{MRR*}>\pi_{T}^{N*}>\pi_{T}^{RR*}$ and $\pi_{T}^{C*}>\pi_{T}^{MR*}>\pi_{T}^{N*}>\pi_{T}^{MRR*}>\pi_{T}^{RR*}$ as the competitive intensity θ increases. The vertical cooperation of the manufacturer and the retailers is conducive to improving the profits of the entire supply chain, but the retailers’ overall situation need to be enhanced under a higher competitive intensity scenario.

The optimal decision results of the manufacturer and the retailer, the optimal market demands and profits generally increase with competitive intensity θ.

## 7. Conclusions

In this study, we have proposed a CLSC model with a manufacturer and two retailers, and the market demand considers the impact of price competition, the carbon emission reduction level and the low-carbon promotion effort competition. We compare five different models, which are the completely decentralized model, M-R vertical cooperation model, R-R horizontal cooperation model, M-R-R vertical and horizontal cooperation model and completely centralized model. We find that the conclusions by systematic comparison are as follows:

In the completely cooperation (C) scenario, the optimal retail price is smallest, and the optimal return rate, the carbon emissions level, the low-carbon promotion effort and the market demand are best among all models.

In the three kinds of semi-cooperation (MR, RR, MRR), more consumers would like to purchase products and the manufacturer would like to increase investment in carbon emissions reduction and collection of used products under the vertical cooperation of manufacturer and retailers (MR), and this cooperation increases the manufacturer’s profit. On the other hand, the retailers’ horizontal cooperation (RR) is passive in the resource recycling, environmental protection and the manufacturer’s profit.

Comparing with the no-cooperation (N), when the competitive intensity is very small, the vertical and horizontal cooperation of the manufacturer and retailers (MRR) is positive, but that is gradually passive with the increasing competition intensity. From the numerical experiment, we came to the following conclusions: the ordinal relationship of the wholesale price and retailers’ profit show diversity. The profit of the entire supply chain is best under the complete cooperation model (C). The vertical cooperation of manufacturer and retailers is conducive to improving the profits of the entire supply chain, but the retailers’ overall situation need to be enhanced under higher competitive intensity conditions.

## Figures and Tables

**Table 1:** Model Notations.

**Model Parameters**	
a	The basic market demand
λ	The consumer’s low-carbon preferences
γ	The consumer’s promotion preferences
θ	The two retailers’ competitive intensity
$c_{r}$	The unit cost of producing end products from used-products
$c_{m}$	The unit cost of producing end products from new materials
δ	The unit cost savings for the manufacturer through remanufacturing
$C_{L}$	The effective investment coefficient of manufacturer and its corresponding used-product collection rate
I	The used-product collection investment of the collecting channel manufacturer
U	The carbon emission reduction effort cost coefficient
K	The low-carbon promotion effort cost coefficient
ξ	The subsidy rate
**Decision variables**	
w	The manufacturer’s wholesale price
$p_{x}$	The retail price of retailer x
τ	The return rate of used products
e	The carbon emission reduction level in the producing end products process
$\upsilon_{x}$	The low-carbon promotion effort in process of retailer x selling products
**Other Notations**	
$d_{x}$	The market demands
$\pi_{f}^{o}$	The profit function for supply chain member f in model o

**Table 2:** The influence of competition intensity on the manufacturer’s decision results.

Competitive Intensity θ								
Decision results	θ values	0	0.1	0.2	0.3	0.4	0.5	0.6
Wholesale price	$w^{N*}$	33.404	36.252	39.938	44.870	51.778	62.082	78.987
	$w^{MR*}$	34.155	37.098	40.893	45.954	53.020	63.528	80.728
	$w^{RR*}$	33.404	36.363	40.121	45.042	51.748	61.406	76.488
	$w^{MRR*}$	34.155	37.149	40.945	45.904	52.652	62.361	77.509
Carbon emission reduction level	$e^{N*}$	0.3755	0.4626	0.5769	0.7317	0.9509	1.2810	1.8274
	$e^{MR*}$	0.3932	0.4830	0.6004	0.7589	0.9827	1.3190	1.8743
	$e^{RR*}$	0.3755	0.4291	0.4961	0.5825	0.6990	0.8653	1.1235
	$e^{MRR*}$	0.3932	0.4466	0.5133	0.5996	0.7161	0.8825	1.1411
	$e^{C*}$	0.9772	1.0915	1.2387	1.4347	1.7077	2.1136	2.7793
Used‐products return rate	${𝜏}^{N*}$	0.3379	0.3747	0.4154	0.4610	0.5135	0.5765	0.6578
	${𝜏}^{MR*}$	0.3539	0.3912	0.4323	0.4781	0.5307	0.5936	0.6748
	${𝜏}^{RR*}$	0.3379	0.3476	0.3572	0.3670	0.3775	0.3894	0.4045
	${𝜏}^{MRR*}$	0.3539	0.3617	0.3696	0.3778	0.3867	0.3971	0.4108
	${𝜏}^{C*}$	0.8795	0.8841	0.8919	0.9038	0.9221	0.9511	1.0000

**Table 3:** The influence of competition intensity on the retailer’s decision results.

Competitive Intensity *θ*								
Decision results	θ values	0	0.1	0.2	0.3	0.4	0.5	0.6
Retail price	$p_{x}^{N*}$	44.668	48.744	53.783	60.236	68.893	81.297	100.92
	$p_{x}^{MR*}$	45.125	49.226	54.293	60.776	69.470	81.929	101.65
	$p_{x}^{RR*}$	44.668	49.237	55.004	62.518	72.718	87.366	110.19
	$p_{x}^{MRR*}$	45.125	49.703	55.483	63.012	73.232	87.910	110.78
	$p_{x}^{C*}$	36.124	39.484	43.783	49.482	57.399	69.141	88.370
Low-carbon promotion effort	$\upsilon_{x}^{N*}$	0.6758	0.7495	0.8307	0.9219	1.0269	1.1529	1.3157
	$\upsilon_{x}^{MR*}$	0.8257	0.9129	1.0086	1.1156	1.2382	1.3850	1.5744
	$\upsilon_{x}^{RR*}$	0.6758	0.6952	0.7144	0.7340	0.7549	0.7788	0.8089
	$\upsilon_{x}^{MRR*}$	0.8257	0.8441	0.8624	0.8815	0.9022	0.9266	0.9585
	$\upsilon_{x}^{C*}$	1.7590	1.7683	1.7837	1.8077	1.8443	1.9023	2.0011

**Table 4:** The influence of competition intensity on the market demand.

Competitive Intensity *θ*								
Demand	θ values	0	0.1	0.2	0.3	0.4	0.5	0.6
Market Demand	$d^{N*}$	22.528	24.982	27.691	30.731	34.231	38.431	43.857
	$d^{MR*}$	23.591	26.082	28.818	31.874	35.378	39.571	44.984
	$d^{RR*}$	22.528	23.173	23.812	24.466	25.164	25.960	26.963
	$d^{MRR*}$	23.591	24.116	24.641	25.185	25.778	26.476	27.385
	$d^{C*}$	58.632	58.942	59.458	60.256	61.478	63.408	66.702

**Table 5:** The influence of competition intensity on the profits.

Competitive Intensity *θ*								
Profits	θ values	0	0.1	0.2	0.3	0.4	0.5	0.6
Profits of manufacturers	$\pi_{M}^{N*}$	337.92	444.13	588.44	790.23	1083.98	1537.23	2302.47
	$\pi_{M}^{MR*}$	353.87	463.68	612.38	819.61	1120.31	1582.85	2361.66
	$\pi_{M}^{RR*}$	337.92	411.97	506.01	629.12	796.85	1038.40	1415.58
	$\pi_{M}^{MRR*}$	353.87	428.73	523.61	647.60	816.31	1059.02	1437.73
Profits of retailer	$\pi_{Rx}^{N*}$	104.04	127.94	157.19	193.60	240.21	302.77	394.30
	$\pi_{Rx}^{MR*}$	105.05	128.40	156.75	191.76	236.24	295.56	381.95
	$\pi_{Rx}^{RR*}$	104.04	125.00	151.68	186.84	235.34	306.63	421.68
	$\pi_{Rx}^{MRR*}$	105.05	125.93	152.55	187.67	236.18	307.54	422.79
Profits of entire supply chain	$\pi_{T}^{N*}$	546.01	700.02	902.82	1177.43	1564.39	2142.77	3091.10
	$\pi_{T}^{MR*}$	563.96	720.48	925.87	1203.13	1592.79	2173.98	3125.55
	$\pi_{T}^{RR*}$	546.01	661.97	809.37	1002.79	1267.54	1651.67	2258.93
	$\pi_{T}^{MRR*}$	563.96	680.60	828.71	1022.95	1288.67	1674.10	2283.31
	$\pi_{T}^{C*}$	879.48	1047.86	1263.48	1549.44	1946.79	2536.33	3501.85

## Appendix A

### Appendix A.1. N Model

Concavity plays a key role in the deduction of optimal strategies. For this reason, we first prove the concavity of $\pi_{R_{x}}^{N}$.

The retailers’ problem: the Hessian matrix of $\pi_{Rx}^{N}$ with respect to $p_{x}$ and $\upsilon_{x}$ is:

$$\pi_{Rx}^{N}=\left(\begin{array}{cc} \begin{array}{c} -2\\ \gamma \end{array} & \begin{array}{c} \gamma \\ -K \end{array} \end{array}\right)$$

When $2K-\gamma^{2}>0$, $\;\pi_{R_{x}}^{N}$ is concave with respect to $p_{x}$ and $\upsilon_{x}$.

By solving the following equations:

$$\{\begin{array}{c} \frac{\;\partial \;\pi_{R_{x}}^{N}}{\partial p_{x}}=0\\ \frac{\;\partial \;\pi_{R_{x}}^{N}}{\partial \upsilon_{x}}=0 \end{array}$$

We obtain the optimal response functions of the retailer for given w and e.

$$p_{x}^{N}\left(w,e\right)=\frac{K\left(a+e\lambda \right)+w\left[K-\gamma^{2}\left(1-\theta \right)\right]}{K\left(2-\theta \right)-\gamma^{2}\left(1-\theta \right)}$$

$$\upsilon_{x}^{N}\left(w,e\right)=\frac{\gamma \left[a-w\left(1-\theta \right)+e\lambda \right]}{K\left(2-\theta \right)-\gamma^{2}\left(1-\theta \right)}$$

Substituting $p_{x}^{N}\left(w,e\right)$ and $\upsilon_{x}^{N}\left(w,e\right)$ into Equation (1). The manufacturer’s problem: the Hessian matrix of $\pi_{M}^{N}$ with respect to w, τ and e is:

When $\left[K\left(2-\theta \right)-\gamma^{2}\left(1-\theta \right)\right]C_{L}-K\delta^{2}\left(1-\theta \right)>0$ and $\left\{K\left[U\left(2-3\theta +\theta^{2}\right)-\lambda^{2}\right]-U\gamma^{2}{\left(1-\theta \right)}^{2}\right\}C_{L}-KU\delta^{2}{\left(1-\theta \right)}^{2}>0$, $\pi_{M}^{N}$ is concave with respect to w, τ and e. From the first-order conditions, we get $w^{N*}$, $\tau^{N*}$, $e^{N*}$. Substituting $w^{N*}$ and $e^{N*}$ into $p_{x}^{N}\left(w,e\right)$ and $\upsilon_{x}^{N}\left(w,e\right)$, we get $p_{x}^{N*}$ and $\upsilon_{x}^{N*}$.

### Appendix A.2. MR Model

Concavity plays a key role in the deduction of optimal strategies. For this reason, we first prove the concavity of $\;\pi_{R_{x}}^{MR}$.

The retailers’ problem: the Hessian matrix of $\pi_{Rx}^{MR}$ with respect to $p_{x}$ and $\upsilon_{x}$ is:

$$\pi_{R_{x}}^{MR}=\left(\begin{array}{cc} \begin{array}{c} -2\\ \gamma -\xi \end{array} & \begin{array}{c} \gamma -\xi \\ -K+2\gamma \xi \end{array} \end{array}\right)$$

When $2K-{\left(\gamma +\xi \right)}^{2}>0$, $\pi_{R_{x}}^{MR}$ is concave with respect to $p_{x}$ and $\upsilon_{x}$.

By solving the following equations:

$$\{\begin{array}{c} \frac{\partial \;\pi_{R_{x}}^{MR}}{\partial p_{x}}=0\\ \frac{\partial \;\pi_{R_{x}}^{MR}}{\partial \upsilon_{x}}=0 \end{array}$$

We obtain the optimal response functions of the retailer for given w and e:

$$p_{x}^{MR}\left(w,e\right)=\frac{\left(K-\gamma \xi -\xi^{2}\right)\left(a+e\lambda \right)+w\left[K-\gamma^{2}\left(1-\theta \right)-\gamma \left(1-\theta \right)\xi \right]}{K\left(2-\theta \right)-\left(1-\theta \right){\left(\gamma +\xi \right)}^{2}}$$

$$\upsilon_{x}^{MR}\left(w,e\right)=\frac{\left[a-w\left(1-\theta \right)+e\lambda \right]\left(\gamma +\xi \right)}{K\left(2-\theta \right)-\left(1-\theta \right){\left(\gamma +\xi \right)}^{2}}$$

Substituting $p_{x}^{MR}\left(w,e\right)$ and $\upsilon_{x}^{MR}\left(w,e\right)$ into Equation (3). The manufacturer’s problem: the Hessian matrix of $\pi_{M}^{MR}$ with respect to w, τ and e is:

When $\left[K\left(2-\theta \right)-\gamma \left(1-\theta \right)\left(\gamma +\xi \right)\right]C_{L}-K\delta^{2}\left(1-\theta \right)>0$ and $\left\{K\left[U\left(2-3\theta +\theta^{2}\right)-\lambda^{2}\right]-U\gamma {\left(1-\theta \right)}^{2}\left(\gamma +\xi \right)\right\}C_{L}-KU\delta^{2}{\left(1-\theta \right)}^{2}>0$, $\pi_{M}^{MR}$ is concave with respect to w, τ and e. From the first-order conditions, we get $w^{MR*}$, $\tau^{MR*}$, $e^{MR*}$. Substituting $w^{MR*}$ and $e^{MR*}$ into $p_{x}^{MR}\left(w,e\right)$ and $\upsilon_{x}^{MR}\left(w,e\right)$, we get $p_{x}^{MR*}$ and $\upsilon_{x}^{MR*}$.

### Appendix A.3. RR Model

Concavity plays a key role in the deduction of optimal strategies. For this reason, we first prove the concavity of $\pi_{R}^{RR}$.

The retailers’ problem: the Hessian matrix of $\pi_{R}^{RR}$ with respect to $p_{x}$ and $\upsilon_{x}$ is:

$$\pi_{R}^{RR}=\left(\begin{array}{cc} \begin{array}{c} \begin{array}{cc} -2 & 2\theta \\ 2\theta & -2 \end{array}\\ \begin{array}{cc} \gamma & -\gamma \theta \\ -\gamma \theta & \gamma \end{array} \end{array} & \begin{array}{c} \begin{array}{cc} \gamma & -\gamma \theta \\ -\gamma \theta & \gamma \end{array}\\ \begin{array}{cc} -K & 0\\ 0 & -K \end{array} \end{array} \end{array}\right)$$

When $4K\left(K-\gamma^{2}\right)+\gamma^{4}\left(1-\theta^{2}\right)>0$, $\pi_{R}^{RR}$ is concave with respect to $p_{x}$ and $\upsilon_{x}$.

By solving the following equations:

$$\{\begin{array}{c} \frac{\partial \pi_{R}^{RR}}{\partial p_{x}}=0\\ \frac{\partial \pi_{R}^{RR}}{\partial \upsilon_{x}}=0 \end{array}$$

We obtain the optimal response functions of the retailer for given w and e.

$$p_{x}^{RR}\left(w,e\right)=\frac{K\left[a+w\left(1-\theta \right)+e\lambda \right]-w\gamma^{2}{\left(1-\theta \right)}^{2}}{\left(2K-\gamma^{2}\left(1-\theta \right)\right)\left(1-\theta \right)}$$

$$\upsilon_{x}^{RR}\left(w,e\right)=\frac{\gamma \left[a-w\left(1-\theta \right)+e\lambda \right]}{2K-\gamma^{2}\left(1-\theta \right)}$$

Substituting $p_{x}^{RR}\left(w,e\right)$ and $\upsilon_{x}^{RR}\left(w,e\right)$ into Equation (5). The manufacturer’s problem: the Hessian matrix of $\pi_{M}^{RR}$ with respect to w, τ and e is:

When $\left[2K-\gamma^{2}\left(1-\theta \right)\right]C_{L}-K\delta^{2}\left(1-\theta \right)>0$ and $\left\{K\left[2U\left(1-\theta \right)-\lambda^{2}\right]-U\gamma^{2}{\left(1-\theta \right)}^{2}\right\}C_{L}-KU\delta^{2}{\left(1-\theta \right)}^{2}>0$, $\pi_{M}^{RR}$ is concave with respect to w, τ and e. From the first-order conditions, we get $w^{RR*}$, $\tau^{RR*}$, $e^{RR*}$. Substituting $w^{RR*}$ and $e^{RR*}$ into $p_{x}^{RR}\left(w,e\right)$ and $\upsilon_{x}^{RR}\left(w,e\right)$, we get $p_{x}^{RR*}$ and $\upsilon_{x}^{RR*}$.

### Appendix A.4. MRR Model

Concavity plays a key role in the deduction of optimal strategies. For this reason, we first prove the concavity of $\pi_{R}^{MRR}$.

The retailers’ problem: the Hessian matrix of $\pi_{R}^{RR}$ with respect to $p_{x}$ and $\upsilon_{x}$ is:

$$\pi_{R}^{MRR}=\left(\begin{array}{cc} \begin{array}{c} \begin{array}{cc} -2 & 2\theta \\ 2\theta & -2 \end{array}\\ \begin{array}{cc} \gamma -\xi & -\theta \left(\gamma -\xi \right)\\ -\theta \left(\gamma -\xi \right) & \gamma -\xi \end{array} \end{array} & \begin{array}{c} \begin{array}{cc} \gamma -\xi & -\theta \left(\gamma -\xi \right)\\ -\theta \left(\gamma -\xi \right) & \gamma -\xi \end{array}\\ \begin{array}{cc} -K+2\gamma \xi & -2\gamma \theta \xi \\ -2\gamma \theta \xi & -K+2\gamma \xi \end{array} \end{array} \end{array}\right)$$

When $4K\left[K-{\left(\gamma +\xi \right)}^{2}\right]+{\left(\gamma +\xi \right)}^{4}\left(1-\theta^{2}\right)>0$, $\pi_{R}^{MRR}$ is concave with respect to $p_{x}$ and $\upsilon_{x}$.

By solving the following equations:

$$\{\begin{array}{c} \frac{\partial \pi_{R}^{MRR}}{\partial p_{x}}=0\\ \frac{\partial \pi_{R}^{MRR}}{\partial \upsilon_{x}}=0 \end{array}$$

We obtain the optimal response functions of the retailer for given w and e.

$$p_{x}^{MRR}\left(w,e\right)=\frac{\left[K-\left(1-\theta \right)\xi \left(\gamma +\xi \right)]\left(a+e\lambda \right)+w\left(1-\theta \right)[K-\gamma \left(1-\theta \right)\left(\gamma +\xi \right)\right]}{\left(1-\theta \right)\left[2K-\left(1-\theta \right){\left(\gamma +\xi \right)}^{2}\right]}$$

$$\upsilon_{x}^{MRR}\left(w,e\right)=\frac{\left[a-w\left(1-\theta \right)+e\lambda \right]\left(\gamma +\xi \right)}{2K-\left(1-\theta \right){\left(\gamma +\xi \right)}^{2}}$$

Substituting $p_{x}^{MRR}\left(w,e\right)$ and $\upsilon_{x}^{MRR}\left(w,e\right)$ into Equation (7). The manufacturer’s problem: the Hessian matrix of $\pi_{M}^{MRR}$ with respect to w, τ and e is:

When $\left[2K-\gamma \left(1-\theta \right)\left(\gamma +\xi \right)\right]C_{L}-K\delta^{2}\left(1-\theta \right)>0$ and $\left\{K\left[2U\left(1-\theta \right)-\lambda^{2}\right]-U\gamma {\left(1-\theta \right)}^{2}\left(\gamma +\xi \right)\right\}C_{L}-KU\delta^{2}{\left(1-\theta \right)}^{2}>0$, $\pi_{M}^{MRR}$ is concave with respect to w, τ and e. From the first-order conditions, we get $w^{MRR*}$, $\tau^{MRR*}$, $e^{MRR*}$. Substituting $w^{MRR*}$ and $e^{MRR*}$ into $p_{x}^{MRR}\left(w,e\right)$ and $\upsilon_{x}^{MRR}\left(w,e\right)$, we get $p_{x}^{MRR*}$ and $\upsilon_{x}^{MRR*}$.

### Appendix A.5. C Model

Concavity plays a key role in the deduction of optimal strategies. For this reason, we prove the concavity of $\pi_{T}^{C}$.

The entire supply chain’s problem: the Hessian matrix of $\pi_{T}^{C}$ with respect to $p_{x}$,  τ, e and $\upsilon_{x}$ is:

$$\pi_{T}^{C}=\left(\begin{array}{cc} \begin{array}{ccc} -2 & 2\theta & \gamma \\ 2\theta & -2 & -\gamma \theta \\ \gamma & -\gamma \theta & -K \end{array} & \begin{array}{ccc} -\gamma \theta & -\delta \left(1-\theta \right) & \lambda \\ \gamma & -\delta \left(1-\theta \right) & \lambda \\ 0 & \gamma \delta \left(1-\theta \right) & 0 \end{array}\\ \begin{array}{ccc} -\gamma \theta & \gamma & 0\\ -\delta \left(1-\theta \right) & -\delta \left(1-\theta \right) & \gamma \delta \left(1-\theta \right)\\ \lambda & \lambda & 0 \end{array} & \begin{array}{ccc} -K & \gamma \delta \left(1-\theta \right) & 0\\ \gamma \delta \left(1-\theta \right) & -C_{L} & 2\delta \lambda \\ 0 & 2\delta \lambda & -U \end{array} \end{array}\right)$$

When $2K-\gamma^{2}\left(1+\theta \right)>0$ and $\left[2K-\gamma^{2}\left(1-\theta \right)\right]C_{L}-2K\delta^{2}\left(1-\theta \right)>0$, $\pi_{T}^{C}$ is concave with respect to $p_{x}$, τ, e and $\upsilon_{x}$.

From the first-order conditions, we get $p_{x}^{C*}$, $\tau^{C*}$, $e^{C*}$, $\upsilon_{x}^{C*}$.

## Appendix B

**Proof of Theorem** **1.** It is easy to prove that:

$$\frac{\partial w^{N*}}{\partial \lambda }=\frac{KU\lambda \left[a-\left(1-\theta \right)c_{m}\right]C_{L}\left\{\left[K\left(2-\theta \right)-\gamma^{2}\left(1-\theta \right)\right]C_{L}-2K\delta^{2}\left(1-\theta \right)\right\}}{{\left\{\left[KU\left(2-3\theta +\theta^{2}\right)-K\lambda^{2}-U\gamma^{2}{\left(1-\theta \right)}^{2}\right]C_{L}-KU\delta^{2}{\left(1-\theta \right)}^{2}\right\}}^{2}}>0$$

$$\frac{\partial w^{N*}}{\partial \gamma }=\frac{KU\gamma \left(1-\theta \right)\left[a-\left(1-\theta \right)c_{m}\right]C_{L}\left[\lambda^{2}C_{L}-U\delta^{2}{\left(1-\theta \right)}^{2}\right]}{{\left\{\left[KU\left(2-3\theta +\theta^{2}\right)-K\lambda^{2}-U\gamma^{2}{\left(1-\theta \right)}^{2}\right]C_{L}-KU\delta^{2}{\left(1-\theta \right)}^{2}\right\}}^{2}}>0$$

$$\frac{\partial \tau^{N*}}{\partial \lambda }=\frac{2K^{2}U\delta \left(1-\theta \right)\lambda \left[a-\left(1-\theta \right)c_{m}\right]C_{L}}{{\left\{\left[KU\left(2-3\theta +\theta^{2}\right)-K\lambda^{2}-U\gamma^{2}{\left(1-\theta \right)}^{2}\right]C_{L}-KU\delta^{2}{\left(1-\theta \right)}^{2}\right\}}^{2}}>0$$

$$\frac{\partial \tau^{N*}}{\partial \gamma }=\frac{2KU^{2}\gamma \delta {\left(1-\theta \right)}^{3}\left[a-\left(1-\theta \right)c_{m}\right]C_{L}}{{\left\{\left[KU\left(2-3\theta +\theta^{2}\right)-K\lambda^{2}-U\gamma^{2}{\left(1-\theta \right)}^{2}\right]C_{L}-KU\delta^{2}{\left(1-\theta \right)}^{2}\right\}}^{2}}>0$$

$$\frac{\partial e^{N*}}{\partial \lambda }=\frac{K\left[a-\left(1-\theta \right)c_{m}\right]C_{L}\left\{\left[KU\left(2-3\theta +\theta^{2}\right)+K\lambda^{2}-U\gamma^{2}{\left(1-\theta \right)}^{2}\right]C_{L}-KU\delta^{2}{\left(1-\theta \right)}^{2}\right\}}{{\left\{\left[KU\left(2-3\theta +\theta^{2}\right)-K\lambda^{2}-U\gamma^{2}{\left(1-\theta \right)}^{2}\right]C_{L}-KU\delta^{2}{\left(1-\theta \right)}^{2}\right\}}^{2}}>0$$

$$\frac{\partial e^{N*}}{\partial \gamma }=\frac{2KU\gamma {\left(1-\theta \right)}^{2}\lambda \left[a-\left(1-\theta \right)c_{m}\right]C_{L}^{2}}{{\left\{\left[KU\left(2-3\theta +\theta^{2}\right)-K\lambda^{2}-U\gamma^{2}{\left(1-\theta \right)}^{2}\right]C_{L}-KU\delta^{2}{\left(1-\theta \right)}^{2}\right\}}^{2}}>0;$$

$$\frac{\partial p_{x}^{N*}}{\partial \lambda }=\frac{KU\lambda \left[a-\left(1-\theta \right)c_{m}\right]C_{L}\left\{\left[K\left(3-2\theta \right)-\gamma^{2}\left(1-\theta \right)\right]C_{L}-2K\delta^{2}\left(1-\theta \right)\right\}}{{\left\{\left[KU\left(2-3\theta +\theta^{2}\right)-K\lambda^{2}-U\gamma^{2}{\left(1-\theta \right)}^{2}\right]C_{L}-KU\delta^{2}{\left(1-\theta \right)}^{2}\right\}}^{2}}>0,$$

$$\frac{\partial p_{x}^{N*}}{\partial \gamma }=\frac{KU\gamma \left(1-\theta \right)\left[a-\left(1-\theta \right)c_{m}\right]C_{L}\left\{\left[U{\left(1-\theta \right)}^{2}+\lambda^{2}\right]C_{L}-U\delta^{2}{\left(1-\theta \right)}^{2}\right\}}{{\left\{\left[KU\left(2-3\theta +\theta^{2}\right)-K\lambda^{2}-U\gamma^{2}{\left(1-\theta \right)}^{2}\right]C_{L}-KU\delta^{2}{\left(1-\theta \right)}^{2}\right\}}^{2}}>0;$$

$$\frac{\partial \upsilon_{x}^{N*}}{\partial \lambda }=\frac{KU\gamma \left(1-\theta \right)\lambda \left[a-\left(1-\theta \right)c_{m}\right]C_{L}^{2}}{{\left\{\left[KU\left(2-3\theta +\theta^{2}\right)-K\lambda^{2}-U\gamma^{2}{\left(1-\theta \right)}^{2}\right]C_{L}-KU\delta^{2}{\left(1-\theta \right)}^{2}\right\}}^{2}}>0,$$

$$\frac{\partial \upsilon_{x}^{N*}}{\partial \gamma }=\frac{U\left(1-\theta \right)\left[a-\left(1-\theta \right)c_{m}\right]C_{L}\left\{\left[KU\left(2-3\theta +\theta^{2}\right)-K\lambda^{2}+U\gamma^{2}{\left(1-\theta \right)}^{2}\right]C_{L}-KU\delta^{2}{\left(1-\theta \right)}^{2}\right\}}{2{\left\{\left[KU\left(2-3\theta +\theta^{2}\right)-K\lambda^{2}-U\gamma^{2}{\left(1-\theta \right)}^{2}\right]C_{L}-KU\delta^{2}{\left(1-\theta \right)}^{2}\right\}}^{2}}>0$$

$$\frac{\partial d^{N*}}{\partial \lambda }=\frac{2K^{2}U\left(1-\theta \right)\lambda \left[a-\left(1-\theta \right)c_{m}\right]C_{L}^{2}}{{\left\{\left[KU\left(2-3\theta +\theta^{2}\right)-K\lambda^{2}-U\gamma^{2}{\left(1-\theta \right)}^{2}\right]C_{L}-KU\delta^{2}{\left(1-\theta \right)}^{2}\right\}}^{2}}>0,$$

$$\frac{\partial d^{N*}}{\partial \gamma }=\frac{2KU^{2}\gamma {\left(1-\theta \right)}^{3}\left[a-\left(1-\theta \right)c_{m}\right]C_{L}^{2}}{{\left\{\left[KU\left(2-3\theta +\theta^{2}\right)-K\lambda^{2}-U\gamma^{2}{\left(1-\theta \right)}^{2}\right]C_{L}-KU\delta^{2}{\left(1-\theta \right)}^{2}\right\}}^{2}}>0;$$

$$\frac{\partial \pi_{M}^{N*}}{\partial \lambda }=\frac{K^{2}U\lambda {\left[a-\left(1-\theta \right)c_{m}\right]}^{2}C_{L}^{2}}{{\left\{\left[KU\left(2-3\theta +\theta^{2}\right)-K\lambda^{2}-U\gamma^{2}{\left(1-\theta \right)}^{2}\right]C_{L}-KU\delta^{2}{\left(1-\theta \right)}^{2}\right\}}^{2}}>0,$$

$$\frac{\partial \pi_{M}^{N*}}{\partial \gamma }=\frac{KU^{2}\gamma {\left(1-\theta \right)}^{2}{\left[a-\left(1-\theta \right)c_{m}\right]}^{2}C_{L}^{2}}{{\left\{\left[KU\left(2-3\theta +\theta^{2}\right)-K\lambda^{2}-U\gamma^{2}{\left(1-\theta \right)}^{2}\right]C_{L}-KU\delta^{2}{\left(1-\theta \right)}^{2}\right\}}^{2}}>0;$$

$$\frac{\partial \;\pi_{R_{x}}^{N*}}{\partial \lambda }=\frac{K^{2}U^{2}\left(2K-\gamma^{2}\right){\left(1-\theta \right)}^{2}\lambda {\left[a-\left(1-\theta \right)c_{m}\right]}^{2}C_{L}^{3}}{2{\left\{\left[KU\left(2-3\theta +\theta^{2}\right)-K\lambda^{2}-U\gamma^{2}{\left(1-\theta \right)}^{2}\right]C_{L}-KU\delta^{2}{\left(1-\theta \right)}^{2}\right\}}^{3}}>0,$$

Hence, we have the conclusions of Theorem 1.

**Proof of Theorem** **2.** It is easy to prove that:

$$\frac{\partial \tau^{MR*}}{\partial \xi }=\frac{KU^{2}\gamma \delta {\left(1-\theta \right)}^{3}\left[a-\left(1-\theta \right)c_{m}\right]C_{L}}{{\left\{\left[KU\left(2-3\theta +\theta^{2}\right)-K\lambda^{2}-U\gamma {\left(1-\theta \right)}^{2}\left(\gamma +\xi \right)\right]C_{L}-KU\delta^{2}{\left(1-\theta \right)}^{2}\right\}}^{2}}>0,$$

$$\frac{\partial e^{MR*}}{\partial \xi }=\frac{KU\gamma {\left(1-\theta \right)}^{2}\lambda \left[a-\left(1-\theta \right)c_{m}\right]C_{L}^{2}}{{\left\{\left[KU\left(2-3\theta +\theta^{2}\right)-K\lambda^{2}-U\gamma {\left(1-\theta \right)}^{2}\left(\gamma +\xi \right)\right]C_{L}-KU\delta^{2}{\left(1-\theta \right)}^{2}\right\}}^{2}}>0,$$

$$\frac{\partial \upsilon_{x}^{MR*}}{\partial \xi }=\frac{KU\left(1-\theta \right)\left[a-\left(1-\theta \right)c_{m}\right]C_{L}\left\{\left[U\left(2-3\theta +\theta^{2}\right)-\lambda^{2}\right]C_{L}-U\delta^{2}{\left(1-\theta \right)}^{2}\right\}}{2{\left\{\left[KU\left(2-3\theta +\theta^{2}\right)-K\lambda^{2}-U\gamma {\left(1-\theta \right)}^{2}\left(\gamma +\xi \right)\right]C_{L}-KU\delta^{2}{\left(1-\theta \right)}^{2}\right\}}^{2}}>0,$$

$$\frac{\partial d^{MR*}}{\partial \xi }=\frac{KU^{2}\gamma {\left(1-\theta \right)}^{3}\left[a-\left(1-\theta \right)c_{m}\right]C_{L}^{2}}{{\left\{\left[KU\left(2-3\theta +\theta^{2}\right)-K\lambda^{2}-U\gamma {\left(1-\theta \right)}^{2}\left(\gamma +\xi \right)\right]C_{L}-KU\delta^{2}{\left(1-\theta \right)}^{2}\right\}}^{2}}>0,$$

$$\frac{\partial \pi_{M}^{MR*}}{\partial \xi }=\frac{KU^{2}\gamma {\left(1-\theta \right)}^{2}{\left[a-\left(1-\theta \right)c_{m}\right]}^{2}C_{L}^{2}}{2{\left\{\left[KU\left(2-3\theta +\theta^{2}\right)-K\lambda^{2}-U\gamma {\left(1-\theta \right)}^{2}\left(\gamma +\xi \right)\right]C_{L}-KU\delta^{2}{\left(1-\theta \right)}^{2}\right\}}^{2}}>0.$$

$$\begin{array}{c} \;\pi_{R_{x}}^{MR*}-\;\pi_{R_{x}}^{N*}=\frac{KU^{2}{\left(1-\theta \right)}^{2}\left[2K-{\left(\gamma +\xi \right)}^{2}\right]{\left[a-\left(1-\theta \right)c_{m}\right]}^{2}C_{L}^{2}}{8{\left(\Upsilon^{MR*}\right)}^{2}}\\ -\frac{KU^{2}\left(2K-\gamma^{2}\right){\left(1-\theta \right)}^{2}{\left[a-\left(1-\theta \right)c_{m}\right]}^{2}C_{L}^{2}}{8{\left(\Upsilon^{N*}\right)}^{2}} \end{array}$$

When $0<\xi \leq \xi_{1}$ and $0<\theta \leq \theta_{0}$, $\;\pi_{R_{x}}^{MR*}-\;\pi_{R_{x}}^{N*}>0$; When $0<\xi \leq \xi_{1}$ and $\theta_{0}<\theta <1$, or $\xi >\xi_{1}$, $\pi_{R_{x}}^{MR*}-\;\pi_{R_{x}}^{N*}<0$. Hence, we have the conclusions of Theorem 2.

**Proof of Theorem** **3.** It is easy to prove that:

$$\frac{\partial w^{RR*}}{\partial \lambda }=\frac{KU\lambda \left[a-\left(1-\theta \right)c_{m}\right]C_{L}\left\{\left[2K-\gamma^{2}\left(1-\theta \right)\right]C_{L}-2K\delta^{2}\left(1-\theta \right)\right\}}{{\left\{\left[2KU\left(1-\theta \right)-K\lambda^{2}-U\gamma^{2}{\left(1-\theta \right)}^{2}\right]C_{L}-KU\delta^{2}{\left(1-\theta \right)}^{2}\right\}}^{2}}>0,$$

$$\frac{\partial w^{RR*}}{\partial \gamma }=\frac{KU\gamma \left(1-\theta \right)\left[a-\left(1-\theta \right)c_{m}\right]C_{L}\left[\lambda^{2}C_{L}-U\delta^{2}{\left(1-\theta \right)}^{2}\right]}{{\left\{\left[2KU\left(1-\theta \right)-K\lambda^{2}-U\gamma^{2}{\left(1-\theta \right)}^{2}\right]C_{L}-KU\delta^{2}{\left(1-\theta \right)}^{2}\right\}}^{2}}>0;$$

$$\frac{\partial \tau^{RR*}}{\partial \lambda }=\frac{2K^{2}U\delta \left(1-\theta \right)\lambda \left[a-\left(1-\theta \right)c_{m}\right]C_{L}}{{\left\{\left[2KU\left(1-\theta \right)-K\lambda^{2}-U\gamma^{2}{\left(1-\theta \right)}^{2}\right]C_{L}-KU\delta^{2}{\left(1-\theta \right)}^{2}\right\}}^{2}}>0,$$

$$\frac{\partial \tau^{RR*}}{\partial \gamma }=\frac{2KU^{2}\gamma \delta {\left(1-\theta \right)}^{3}\left[a-\left(1-\theta \right)c_{m}\right]C_{L}}{{\left\{\left[2KU\left(1-\theta \right)-K\lambda^{2}-U\gamma^{2}{\left(1-\theta \right)}^{2}\right]C_{L}-KU\delta^{2}{\left(1-\theta \right)}^{2}\right\}}^{2}}>0;$$

$$\frac{\partial e^{RR*}}{\partial \lambda }=\frac{K\left[a-\left(1-\theta \right)c_{m}\right]C_{L}\left\{\left[2KU\left(1-\theta \right)+K\lambda^{2}-U\gamma^{2}{\left(1-\theta \right)}^{2}\right]C_{L}-KU\delta^{2}{\left(1-\theta \right)}^{2}\right\}}{{\left\{\left[2KU\left(1-\theta \right)-K\lambda^{2}-U\gamma^{2}{\left(1-\theta \right)}^{2}\right]C_{L}-KU\delta^{2}{\left(1-\theta \right)}^{2}\right\}}^{2}}>0,$$

$$\frac{\partial e^{RR*}}{\partial \gamma }=\frac{2KU\gamma {\left(1-\theta \right)}^{2}\lambda \left[a-\left(1-\theta \right)c_{m}\right]C_{L}^{2}}{{\left\{\left[2KU\left(1-\theta \right)-K\lambda^{2}-U\gamma^{2}{\left(1-\theta \right)}^{2}\right]C_{L}-KU\delta^{2}{\left(1-\theta \right)}^{2}\right\}}^{2}}>0;$$

$$\frac{\partial p_{x}^{RR*}}{\partial \lambda }=\frac{KU\lambda \left[a-\left(1-\theta \right)c_{m}\right]C_{L}\left\{\left[3K-\gamma^{2}\left(1-\theta \right)\right]C_{L}-2K\delta^{2}\left(1-\theta \right)\right\}}{{\left\{\left[2KU\left(1-\theta \right)-K\lambda^{2}-U\gamma^{2}{\left(1-\theta \right)}^{2}\right]C_{L}-KU\delta^{2}{\left(1-\theta \right)}^{2}\right\}}^{2}}>0,$$

$$\frac{\partial p_{x}^{RR*}}{\partial \gamma }=\frac{KU\gamma \left(1-\theta \right)\left[a-\left(1-\theta \right)c_{m}\right]C_{L}\left\{\left[U{\left(1-\theta \right)}^{2}+\lambda^{2}\right]C_{L}-U\delta^{2}{\left(1-\theta \right)}^{2}\right\}}{{\left\{\left[2KU\left(1-\theta \right)-K\lambda^{2}-U\gamma^{2}{\left(1-\theta \right)}^{2}\right]C_{L}-KU\delta^{2}{\left(1-\theta \right)}^{2}\right\}}^{2}}>0;$$

$$\frac{\partial \upsilon_{x}^{RR*}}{\partial \lambda }=\frac{KU\gamma \left(1-\theta \right)\lambda \left[a-\left(1-\theta \right)c_{m}\right]C_{L}^{2}}{{\left\{\left[2KU\left(1-\theta \right)-K\lambda^{2}-U\gamma^{2}{\left(1-\theta \right)}^{2}\right]C_{L}-KU\delta^{2}{\left(1-\theta \right)}^{2}\right\}}^{2}}>0,$$

$$\frac{\partial \upsilon_{x}^{RR*}}{\partial \gamma }=\frac{U\left(1-\theta \right)\left[a-\left(1-\theta \right)c_{m}\right]C_{L}\left\{\left[2KU\left(1-\theta \right)-K\lambda^{2}+U\gamma^{2}{\left(1-\theta \right)}^{2}\right]C_{L}-KU\delta^{2}{\left(1-\theta \right)}^{2}\right\}}{2{\left\{\left[2KU\left(1-\theta \right)-K\lambda^{2}-U\gamma^{2}{\left(1-\theta \right)}^{2}\right]C_{L}-KU\delta^{2}{\left(1-\theta \right)}^{2}\right\}}^{2}}>0;$$

$$\frac{\partial d^{RR*}}{\partial \lambda }=\frac{2K^{2}U\left(1-\theta \right)\lambda \left[a-\left(1-\theta \right)c_{m}\right]C_{L}^{2}}{{\left\{\left[2KU\left(1-\theta \right)-K\lambda^{2}-U\gamma^{2}{\left(1-\theta \right)}^{2}\right]C_{L}-KU\delta^{2}{\left(1-\theta \right)}^{2}\right\}}^{2}}>0,$$

$$\frac{\partial d^{RR*}}{\partial \gamma }=\frac{2KU^{2}\gamma {\left(1-\theta \right)}^{3}\left[a-\left(1-\theta \right)c_{m}\right]C_{L}^{2}}{{\left\{\left[2KU\left(1-\theta \right)-K\lambda^{2}-U\gamma^{2}{\left(1-\theta \right)}^{2}\right]C_{L}-KU\delta^{2}{\left(1-\theta \right)}^{2}\right\}}^{2}}>0;$$

$$\frac{\partial \pi_{M}^{RR*}}{\partial \lambda }=\frac{K^{2}U\lambda {\left[a-\left(1-\theta \right)c_{m}\right]}^{2}C_{L}^{2}}{{\left\{\left[2KU\left(1-\theta \right)-K\lambda^{2}-U\gamma^{2}{\left(1-\theta \right)}^{2}\right]C_{L}-KU\delta^{2}{\left(1-\theta \right)}^{2}\right\}}^{2}}>0$$

$$\frac{\partial \pi_{M}^{RR*}}{\partial \gamma }=\frac{KU^{2}\gamma {\left(1-\theta \right)}^{2}{\left[a-\left(1-\theta \right)c_{m}\right]}^{2}C_{L}^{2}}{{\left\{\left[2KU\left(1-\theta \right)-K\lambda^{2}-U\gamma^{2}{\left(1-\theta \right)}^{2}\right]C_{L}-KU\delta^{2}{\left(1-\theta \right)}^{2}\right\}}^{2}}>0;$$

$$\frac{\partial \;\pi_{R_{x}}^{RR*}}{\partial \lambda }=\frac{K^{2}U^{2}\left[2K-\gamma^{2}\left(1-\theta \right)\right]\left(1-\theta \right)\lambda {\left[a-\left(1-\theta \right)c_{m}\right]}^{2}C_{L}^{3}}{2{\left\{\left[2KU\left(1-\theta \right)-K\lambda^{2}-U\gamma^{2}{\left(1-\theta \right)}^{2}\right]C_{L}-KU\delta^{2}{\left(1-\theta \right)}^{2}\right\}}^{2}}>0,$$

$$\frac{\partial \;\pi_{R_{x}}^{RR*}}{\partial \gamma }=\frac{KU^{2}\gamma {\left(1-\theta \right)}^{2}{\left[a-\left(1-\theta \right)c_{m}\right]}^{2}C_{L}^{2}\left\{KU\delta^{2}{\left(1-\theta \right)}^{2}+\left[2KU\left(1-\theta \right)+K\lambda^{2}-U\gamma^{2}{\left(1-\theta \right)}^{2}\right]C_{L}\right\}}{4{\left\{\left[2KU\left(1-\theta \right)-K\lambda^{2}-U\gamma^{2}{\left(1-\theta \right)}^{2}\right]C_{L}-KU\delta^{2}{\left(1-\theta \right)}^{2}\right\}}^{3}}>0.$$

Hence, we have the conclusions of Theorem 3.

**Proof of Theorem** **4.** It is easy to prove that:

$$\frac{\partial \tau^{MRR*}}{\partial \xi }=\frac{KU^{2}\gamma \delta {\left(1-\theta \right)}^{3}\left[a-\left(1-\theta \right)c_{m}\right]C_{L}}{{\left\{\left[2KU\left(1-\theta \right)-K\lambda^{2}-U\gamma {\left(1-\theta \right)}^{2}\left(\gamma +\xi \right)\right]C_{L}-KU\delta^{2}{\left(1-\theta \right)}^{2}\right\}}^{2}}>0,$$

$$\frac{\partial e^{MRR*}}{\partial \xi }=\frac{KU\gamma {\left(1-\theta \right)}^{2}\lambda \left[a-\left(1-\theta \right)c_{m}\right]C_{L}^{2}}{{\left\{\left[2KU\left(1-\theta \right)-K\lambda^{2}-U\gamma {\left(1-\theta \right)}^{2}\left(\gamma +\xi \right)\right]C_{L}-KU\delta^{2}{\left(1-\theta \right)}^{2}\right\}}^{2}}>0,$$

$$\frac{\partial \upsilon_{x}^{MRR*}}{\partial \xi }=\frac{KU\left(1-\theta \right)\left[a-\left(1-\theta \right)c_{m}\right]C_{L}\left\{\left[2U\left(1-\theta \right)-\lambda^{2}\right]C_{L}-U\delta^{2}{\left(1-\theta \right)}^{2}\right\}}{2{\left\{\left[2KU\left(1-\theta \right)-K\lambda^{2}-U\gamma {\left(1-\theta \right)}^{2}\left(\gamma +\xi \right)\right]C_{L}-KU\delta^{2}{\left(1-\theta \right)}^{2}\right\}}^{2}}>0,$$

$$\frac{\partial d^{MR*}}{\partial \xi }=\frac{KU^{2}\gamma {\left(1-\theta \right)}^{3}\left[a-\left(1-\theta \right)c_{m}\right]C_{L}^{2}}{{\left\{\left[2KU\left(1-\theta \right)-K\lambda^{2}-U\gamma {\left(1-\theta \right)}^{2}\left(\gamma +\xi \right)\right]C_{L}-KU\delta^{2}{\left(1-\theta \right)}^{2}\right\}}^{2}}>0,$$

$$\frac{\partial \pi_{M}^{MR*}}{\partial \xi }=\frac{KU^{2}\gamma {\left(1-\theta \right)}^{2}{\left[a-\left(1-\theta \right)c_{m}\right]}^{2}C_{L}^{2}}{2{\left\{\left[2KU\left(1-\theta \right)-K\lambda^{2}-U\gamma {\left(1-\theta \right)}^{2}\left(\gamma +\xi \right)\right]C_{L}-KU\delta^{2}{\left(1-\theta \right)}^{2}\right\}}^{2}}>0.$$

$$\begin{array}{c} \;\pi_{R_{x}}^{MRR*}-\;\pi_{R_{x}}^{N*}=\frac{KU^{2}\left(1-\theta \right)\left[2K-\left(1-\theta \right){\left(\gamma +\xi \right)}^{2}\right]{\left[a-\left(1-\theta \right)c_{m}\right]}^{2}C_{L}^{2}}{8{\left(\Upsilon^{MRR*}\right)}^{2}}\\ -\frac{KU^{2}\left(2K-\gamma^{2}\right){\left(1-\theta \right)}^{2}{\left[a-\left(1-\theta \right)c_{m}\right]}^{2}C_{L}^{2}}{8{\left(\Upsilon^{N*}\right)}^{2}}\; \end{array}$$

When $0<\xi \leq \xi_{2}$, $\pi_{R_{x}}^{MRR*}-\;\pi_{R_{x}}^{N*}>0$；When $\xi >\xi_{2}$, $\;\pi_{R_{x}}^{MRR*}-\;\pi_{R_{x}}^{N*}<0$.

Hence, we have the conclusions of Theorem 4.

**Proof of Theorem** **5.** It is easy to prove that:

$$\frac{\partial \tau^{C*}}{\partial \lambda }=\frac{8K^{2}U\delta \left(1-\theta \right)\lambda \left[a-\left(1-\theta \right)c_{m}\right]C_{L}}{{\left\{\left[2KU\left(1-\theta \right)-2K\lambda^{2}-U\gamma^{2}{\left(1-\theta \right)}^{2}\right]C_{L}-2KU\delta^{2}{\left(1-\theta \right)}^{2}\right\}}^{2}}>0,$$

$$\frac{\partial \tau^{C*}}{\partial \gamma }=\frac{4KU^{2}\gamma \delta {\left(1-\theta \right)}^{3}\left[a-\left(1-\theta \right)c_{m}\right]C_{L}}{{\left\{\left[2KU\left(1-\theta \right)-2K\lambda^{2}-U\gamma^{2}{\left(1-\theta \right)}^{2}\right]C_{L}-2KU\delta^{2}{\left(1-\theta \right)}^{2}\right\}}^{2}}>0;$$

$$\frac{\partial e^{C*}}{\partial \lambda }=\frac{K\left[a-\left(1-\theta \right)c_{m}\right]C_{L}\left\{\left[2KU\left(1-\theta \right)+2K\lambda^{2}-U\gamma^{2}{\left(1-\theta \right)}^{2}\right]C_{L}-2KU\delta^{2}{\left(1-\theta \right)}^{2}\right\}}{{\left\{\left[2KU\left(1-\theta \right)-2K\lambda^{2}-U\gamma^{2}{\left(1-\theta \right)}^{2}\right]C_{L}-2KU\delta^{2}{\left(1-\theta \right)}^{2}\right\}}^{2}}>0,$$

$$\frac{\partial e^{C*}}{\partial \gamma }=\frac{4KU\gamma {\left(1-\theta \right)}^{2}\lambda \left[a-\left(1-\theta \right)c_{m}\right]C_{L}^{2}}{{\left\{\left[2KU\left(1-\theta \right)-2K\lambda^{2}-U\gamma^{2}{\left(1-\theta \right)}^{2}\right]C_{L}-2KU\delta^{2}{\left(1-\theta \right)}^{2}\right\}}^{2}}>0;$$

$$\frac{\partial p_{x}^{C*}}{\partial \lambda }=\frac{4K^{2}U\lambda \left[a-\left(1-\theta \right)c_{m}\right]C_{L}\left[C_{L}-2\delta^{2}\left(1-\theta \right)\right]}{{\left\{\left[2KU\left(1-\theta \right)-2K\lambda^{2}-U\gamma^{2}{\left(1-\theta \right)}^{2}\right]C_{L}-2KU\delta^{2}{\left(1-\theta \right)}^{2}\right\}}^{2}}<0,$$

$$\frac{\partial p_{x}^{C*}}{\partial \gamma }=\frac{2KU^{2}\gamma {\left(1-\theta \right)}^{2}\left[a-\left(1-\theta \right)c_{m}\right]C_{L}\left[C_{L}-2\delta^{2}\left(1-\theta \right)\right]}{{\left\{\left[2KU\left(1-\theta \right)-2K\lambda^{2}-U\gamma^{2}{\left(1-\theta \right)}^{2}\right]C_{L}-2KU\delta^{2}{\left(1-\theta \right)}^{2}\right\}}^{2}}>0;$$

$$\frac{\partial \upsilon_{x}^{C*}}{\partial \lambda }=\frac{4KU\gamma \left(1-\theta \right)\lambda \left[a-\left(1-\theta \right)c_{m}\right]C_{L}^{2}}{{\left\{\left[2KU\left(1-\theta \right)-2K\lambda^{2}-U\gamma^{2}{\left(1-\theta \right)}^{2}\right]C_{L}-2KU\delta^{2}{\left(1-\theta \right)}^{2}\right\}}^{2}}>0,$$

$$\frac{\partial \upsilon_{x}^{C*}}{\partial \gamma }=\frac{U\left(1-\theta \right)\left[a-\left(1-\theta \right)c_{m}\right]C_{L}\left\{\left[U\gamma^{2}{\left(1-\theta \right)}^{2}+2KU\left(1-\theta \right)-2K\lambda^{2}\right]C_{L}-2KU\delta^{2}{\left(1-\theta \right)}^{2}\right\}}{{\left\{\left[2KU\left(1-\theta \right)-2K\lambda^{2}-U\gamma^{2}{\left(1-\theta \right)}^{2}\right]C_{L}-2KU\delta^{2}{\left(1-\theta \right)}^{2}\right\}}^{2}}>0;$$

$$\frac{\partial d^{C*}}{\partial \lambda }=\frac{8K^{2}U\left(1-\theta \right)\lambda \left[a-\left(1-\theta \right)c_{m}\right]C_{L}^{2}}{{\left\{\left[2KU\left(1-\theta \right)-2K\lambda^{2}-U\gamma^{2}{\left(1-\theta \right)}^{2}\right]C_{L}-2KU\delta^{2}{\left(1-\theta \right)}^{2}\right\}}^{2}}>0$$

$$\frac{\partial d^{C*}}{\partial \gamma }=\frac{4KU^{2}\gamma {\left(1-\theta \right)}^{3}\left[a-\left(1-\theta \right)c_{m}\right]C_{L}^{2}}{{\left\{\left[2KU\left(1-\theta \right)-2K\lambda^{2}-U\gamma^{2}{\left(1-\theta \right)}^{2}\right]C_{L}-2KU\delta^{2}{\left(1-\theta \right)}^{2}\right\}}^{2}}>0;$$

$$\frac{\partial \pi_{T}^{C*}}{\partial \lambda }=\frac{4K^{2}U\lambda {\left[a-\left(1-\theta \right)c_{m}\right]}^{2}C_{L}^{2}}{{\left\{\left[2KU\left(1-\theta \right)-2K\lambda^{2}-U\gamma^{2}{\left(1-\theta \right)}^{2}\right]C_{L}-2KU\delta^{2}{\left(1-\theta \right)}^{2}\right\}}^{2}}>0,$$

$$\frac{\partial \pi_{T}^{C*}}{\partial \gamma }=\frac{2KU^{2}\gamma {\left(1-\theta \right)}^{2}{\left[a-\left(1-\theta \right)c_{m}\right]}^{2}C_{L}^{2}}{{\left\{\left[2KU\left(1-\theta \right)-2K\lambda^{2}-U\gamma^{2}{\left(1-\theta \right)}^{2}\right]C_{L}-2KU\delta^{2}{\left(1-\theta \right)}^{2}\right\}}^{2}}>0.$$

Hence, we have the conclusions of Theorem 5.

## Appendix C

**Proof of Proposition** **1.** It is easy to prove that:

Hence, we have $w^{MR*}>w^{N*}$,$\;w^{\mathrm{MRR}}>w^{RR}$; $\tau^{C*}>\tau^{MR*}>\tau^{MRR*}>\tau^{N*}>\tau^{RR*}$,$\;e^{C*}>e^{MR*}>e^{MRR*}>e^{N*}>e^{RR*}$, if $0<\theta <\frac{\gamma \xi }{K+\gamma \xi }$; $\tau^{C*}>\tau^{MR*}>\tau^{N*}>\tau^{MRR*}>\tau^{RR*}$,$\;e^{C*}>e^{MR*}>e^{N*}>e^{MRR*}>e^{RR*}$, if $\frac{\gamma \xi }{K+\gamma \xi }<\theta <1$.

**Proof of Proposition** **2.** It is easy to prove that:

Hence, we have $p_{x}^{MRR*}>p_{x}^{RR*}>p_{x}^{C*}$, $\;p_{x}^{MR*}>p_{x}^{N*}>p_{x}^{C*}$; $\upsilon_{x}^{C*}>\upsilon_{x}^{MR*}>\upsilon_{x}^{\mathrm{MRR}*}>\upsilon_{x}^{N*}>\upsilon_{x}^{RR*}$, if $0<\theta <\frac{2\xi }{\gamma +\xi }$; $\;\upsilon_{x}^{C*}>\upsilon_{x}^{MR*}>\upsilon_{x}^{N*}>\upsilon_{x}^{\mathrm{MRR}*}>\upsilon_{x}^{RR*}$, if $\frac{2\xi }{\gamma +\xi }<\theta <1$.

**Proof of Proposition** **3.** It is easy to prove that:

Hence, we have $d^{C*}>d^{MR*}>d^{MRR*}>d^{N*}>d^{RR*}$, if $0<\theta <\frac{\gamma \xi }{K+\gamma \xi }$; $d^{C*}>d^{MR*}>d^{N*}>d^{MRR*}>d^{RR*}$, if $\frac{\gamma \xi }{K+\gamma \xi }<\theta <1$.

**Proof of Proposition** **4.** It is easy to prove that:

Hence, we have $\pi_{M}^{MR*}>\pi_{M}^{MRR*}>\pi_{M}^{N*}>\pi_{M}^{RR*}$, if $0<\theta <\frac{\gamma \xi }{K+\gamma \xi }$; $\pi_{M}^{MR*}>\pi_{M}^{N*}>\pi_{M}^{MRR*}>\pi_{M}^{RR*}$, if $\frac{\gamma \xi }{K+\gamma \xi }<\theta <1$.
